# Bimetallic Ni-Based Catalysts for CO_2_ Methanation: A Review

**DOI:** 10.3390/nano11010028

**Published:** 2020-12-24

**Authors:** Anastasios I. Tsiotsias, Nikolaos D. Charisiou, Ioannis V. Yentekakis, Maria A. Goula

**Affiliations:** 1 Laboratory of Alternative Fuels and Environmental Catalysis (LAFEC), Department of Chemical Engineering, University of Western Macedonia, GR-50100 Koila, Greece; antsiotsias@uowm.gr (A.I.T.); ncharisiou@uowm.gr (N.D.C.); 2 Laboratory of Physical Chemistry & Chemical Processes, School of Environmental Engineering, Technical University of Crete, GR-73100 Chania, Greece; yyentek@isc.tuc.gr

**Keywords:** CO_2_ methanation, bimetallic catalysts, Ni-based catalysts, promoters, alloy nanoparticles, bimetallic synergy

## Abstract

CO_2_ methanation has recently emerged as a process that targets the reduction in anthropogenic CO_2_ emissions, via the conversion of CO_2_ captured from point and mobile sources, as well as H_2_ produced from renewables into CH_4_. Ni, among the early transition metals, as well as Ru and Rh, among the noble metals, have been known to be among the most active methanation catalysts, with Ni being favoured due to its low cost and high natural abundance. However, insufficient low-temperature activity, low dispersion and reducibility, as well as nanoparticle sintering are some of the main drawbacks when using Ni-based catalysts. Such problems can be partly overcome via the introduction of a second transition metal (e.g., Fe, Co) or a noble metal (e.g., Ru, Rh, Pt, Pd and Re) in Ni-based catalysts. Through Ni-M alloy formation, or the intricate synergy between two adjacent metallic phases, new high-performing and low-cost methanation catalysts can be obtained. This review summarizes and critically discusses recent progress made in the field of bimetallic Ni-M (M = Fe, Co, Cu, Ru, Rh, Pt, Pd, Re)-based catalyst development for the CO_2_ methanation reaction.

## 1. Introduction

During the last hundred years, rapid industrialization and the high energy demands of our society have disrupted the carbon cycle through ever increasing greenhouse gas emissions, and the ramp-up of renewable energy production has yet to offset the negative effects on our planet’s climate and ecosystems [1,2]. However, progress made in hydrogen production technologies through water electrolysis has raised hopes for the utilization of this green fuel that produces no CO_2_ emissions upon its combustion [3], despite the fact that its storage and transportation remain challenging when compared to other traditional energy carriers, such as natural gas [4]. In the last decade, research efforts have been focused on the development of catalysts that can utilize this excess renewable hydrogen in order to hydrogenate CO_2_ released from industrial flue gases. This way, H_2_ can be transformed into a reliable energy carrier, that is, CH_4_ or synthetic natural gas (SNG), with a significantly higher energy density, all the while creating a closed carbon cycle [5]. The complete hydrogenation of CO_2_ into CH_4_, or CO_2_ methanation, is also known as the Sabatier reaction and is an exothermic reaction with the following equation:

CO_2_ + 4H_2_ → CH_4_ + 2H_2_O   ΔΗ_298 K_ = −165 kJ/mol (1)

**Figure 1:**
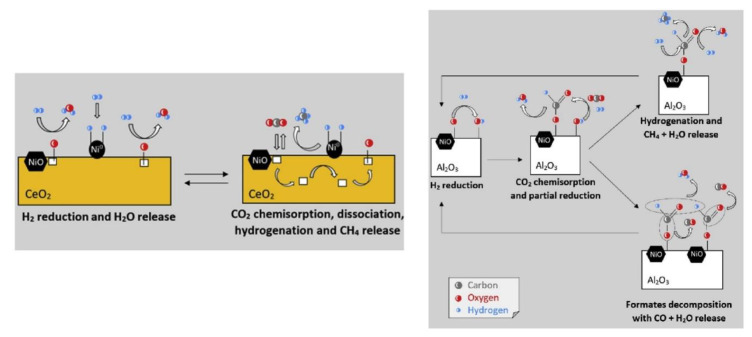
Scheme of the CO_2_ + H_2_ reaction mechanisms over Ni/CeO_2_ and Ni/Al_2_O_3_ catalysts. Reproduced with permission from [12]. Copyright: Elsevier, Amsterdam, The Netherlands, 2020.

Ni has become a favourite active metal for this reaction, since its high methanation activity, low cost and natural abundance render it attractive for industrial-scale applications [6]. Since CH_4_ yield peaks at a relatively low temperature (300–400 °C, depending on the reaction conditions) [7], structural degradation of Ni-based catalysts, though not completely avoided, plays a minor role compared to other reactions (e.g., methane dry reforming) [8]. The choice of the metal oxide support also appears to be of great importance in the performance of Ni-based catalysts [9,10,11]. Ni/CeO_2_ catalysts, for example, are much more active compared to Ni/Al_2_O_3_ or Ni/SiO_2_ catalysts. This is mainly attributed to ceria’s intricate redox and O^2−^-defect chemistry, with it being able to transport oxygen species and oxygen ion vacancies throughout its lattice, having higher basicity compared to other metal oxides that favours CO_2_ chemisorption and activation, as well as exhibiting a strong metal–support interaction that favours a higher Ni dispersion (Figure 1) [12].

The activity of Ni-based catalysts can be further improved via modification of the metal oxide supports. For example, alkali and alkaline earth metals [13], transition metals [6] and rare-earth metals [14] can be used as promoters that modify the physicochemical properties of metal oxide supports. In some cases, these ions can enter the lattice of the metal oxide supports (e.g., Ca^2+^ ions in CeO_2_ and ZrO_2_ lattices) [15], or form segregated metal oxide phases supported on the support surface (e.g., La_2_O_3_, CeO_2_ and MnO_x_ in Al_2_O_3_) [16]. Such modifications can lead to an increase in support basicity, so that the initial step of CO_2_ chemisorption step is accelerated, or to an increase in the active metal dispersion [17]. In most cases, the low-temperature activity and stability of Ni-based catalysts is enhanced following modification of the metal oxide supports [13,14,16].

Besides Ni, Ru and Rh noble metals have been extensively studied as active metallic phases in CO_2_ methanation and they usually achieve a much higher activity at low temperatures [18,19]. Since CH_4_ is thermodynamically favoured over other CO_2_ hydrogenation products such as CO, at low temperatures, CH_4_ selectivity can be significantly higher when using noble metal catalysts [7]. Among the two noble metals, Ru can achieve higher activity and its price is considerably lower compared to Rh, while it can also provide significant methanation activity when supported on cheap supports (e.g., Al_2_O_3_ or TiO_2_) at a metal loading as low as 1% or even 0.5% [20]. Ru is also preferable to Ni for application in the combined capture and methanation of CO_2_ derived from industrial flue-gases since the high reducibility of RuO_x_ oxides allows for isothermal operation at low temperatures [21,22].

A popular method to counter some of the drawbacks of Ni-based catalysts is to use a second metal (e.g., Fe, Co or Ru) as a dopant, in order to create appropriate bimetallic CO_2_ methanation catalysts [6]. Such an approach has been successfully employed in other reactions. For example, NiFe alloys are active and stable catalysts for dry reforming of methane, since Fe can promote carbon gasification and significantly reduce coking through an intricate dealloying and realloying mechanism [23]. The combination of Ni with other metals can either lead to the formation of Ni-M alloys, or monometallic heterostructures with closely located active metallic Ni-M phases [23,24]. There are two types of metals that are used in such Ni-M bimetallic catalysts, the one an early transition metal such as Fe and Co and the other a noble metal, namely Ru, Rh, Pt, Pd and Re.

Fe and Co can easily dissolve into the Ni lattice due to the similar crystallographic properties of the corresponding metallic phases. In the example of Fe, the dissolution of Fe atoms into the Ni lattice leads to the formation of NiFe alloys, with Ni_3_Fe being the most thermodynamically stable [25,26]. The introduction of Fe causes an expansion of the Ni fcc lattice up to a specific Fe amount and a shift of the (111) Ni reflection in XRD towards lower 2θ values. At higher Fe contents, the lattice becomes Fe rich and switches to the more compact bcc structure of pure Fe [27]. The introduction of the dopant metal can be used to tailor the electronic properties of Ni, so that the new alloy phase can achieve superior activity compared to monometallic Ni. This can also lead to a higher dispersion, stability and/or resistance towards deactivation [24]. The application of computational methods has shown that specific alloys can lower the M-CO binding energy and lead to higher CO methanation activities [28].

Noble metals Ru, Rh, Pt, Pd and Re can increase the reaction activity by enhancing the reducibility of the primary Ni phase, by increasing the Ni dispersion, or by changing the reaction pathway [29]. Ru and Ni mostly form monometallic heterostructures that rely on the synergistic effect between the two separate metallic phases, while Pt and Pd mostly lead to the creation of NiPt and NiPd alloys [30,31,32]. It has been shown that an addition of only a miniscule amount of noble metal (e.g., 0.5% or 1%) can greatly enhance the reducibility and low-temperature activity of Ni-based catalysts without the need to use high loadings of precious metals [33].

In this review, we aim to summarize the most recent progress made in the field of Ni-based bimetallic catalyst development for the CO_2_ methanation reaction. Due to the different nature of the reaction promotion, Ni-based catalysts combined with either early transition metals (Fe and Co), or noble metals (Ru, Rh, Pt, Pd and Re), will be discussed in separate chapters.

## 2. Promotion with Transition Metals

There are many works that use a transition metal additive to enhance the activity of Ni-based catalysts [24]. These additives may include: V, Cr, Mn, Fe, Co, Y and Zr. Y and Zr, for example, are mostly used as dopants to modify the lattice of the metal oxide support and enhance its defect chemistry. Zr is used to stabilize the CeO_2_ structure and enhance its oxygen vacancies population (i.e., oxygen storage capacity, OSC) [19], while Y can generate oxygen vacancies in ZrO_2_-based supports [34,35]. Mn mostly forms MnO_x_ phases that increase the catalyst basicity and favour CO_2_ chemisorption. All these modifications on the support’s properties can lead to an increase in CO_2_ activation and, thus, Mn, Ce, Zr and Y are often regarded as efficient promoters in CO_2_ methanation [19,34,35,36].

Fe and Co, when combined with Ni-based catalysts, allow the creation of NiFe and NiCo alloys [26,37]. The incorporation of these transition metal dopants into the lattice of the active Ni phase can directly interfere with nickel’s electronic properties and methanation chemistry [38]. This can either lead to an increase in activity and stability or to a complete catalyst deactivation, depending on the Ni/dopant ratio, its degree of metal intermixing and the interaction of both metals with the support [39].

### 2.1. Promotion with Fe

Among all metals, Fe is by far the most studied element in bimetallic Ni-based catalysts for CO_2_ methanation, since it is quite cheap and highly available and it exhibits a high solubility into the Ni lattice, favouring the formation of NiFe alloys [25]. It has been suggested, based on computational screening and catalytic experiments, that alumina supported and Ni-rich NiFe catalysts can improve the rate of CO_2_ conversion, with the optimal Ni/(Ni + Fe) ratio being around 0.7–0.9 [40,41]. Many more works have focused on NiFe alloys prepared with different methods and supported on various metal oxides [42]. Generally, the Ni/Fe ratio in the alloy and the reducibility of the metal-oxide support appear to be the most critical parameters that determine whether Fe will promote or suppress the catalyst’s CO_2_ methanation activity.

Pandey et al. [43] were among the first to perform a systematic study about the Fe promotion in Ni-based methanation catalysts. The optimal active metal content of the catalysts consisted of 75% Ni and 25% Fe. When compared to their monometallic counterparts (Ni and Fe), the bimetallic NiFe alloy catalysts supported on alumina and silica were shown to exhibit higher CH_4_ yields and this enhancement was more apparent in the alumina supported catalysts. This was attributed to the creation of a suitable alloy phase and to the increased CO_2_ chemisorption at unreduced Fe_3_O_4_ sites. The authors then compared the activity of Ni_3_Fe catalysts supported on different metal oxide supports, such as Al_2_O_3_, SiO_2_, ZrO_2_ and TiO_2_, and noted that Al_2_O_3_ supported catalysts yielded the best results and provided the largest promotion due to Fe alloying [42]. Regarding the optimal alloy composition, a statistical technique, namely response surface methodology (RSM), was also used. The model equation predicted that a 32.78% Ni loading and 7.67% Fe loading would lead to the optimal methane yield, and the obtained experimental results confirmed this model prediction [44].

**Figure 2:**
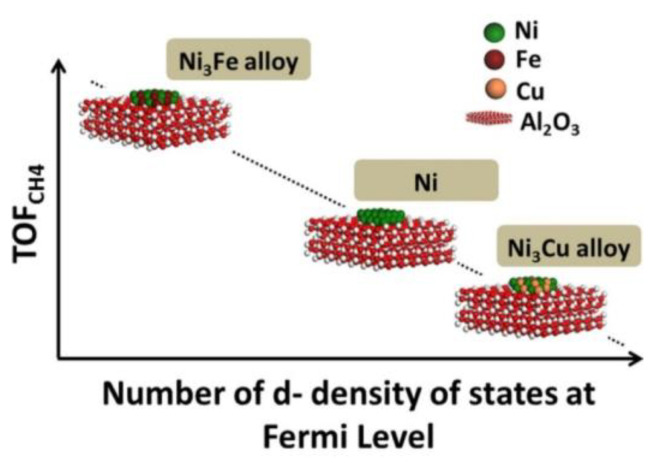
Linear correlation between the turnover frequency for CH_4_ production (TOF_CH4_) and the number of d-density of states (d-DOS) at Fermi level (N_EF_) for Ni, Ni_3_Fe and Ni_3_Cu catalysts. Reproduced with permission from [38]. Copyright: Elsevier, Amsterdam, The Netherlands, 2017.

Ray et al. [38] from the same group managed to develop a descriptor for the methanation of CO_2_ over Ni_3_M bimetallic catalysts, with the second metal (M) being Fe and Cu. It was shown that the turnover frequency for methane production (TOF_CH4_) could be linearly correlated with the number of d-density of states (d-DOS) at the Fermi level (N_EF_) based on density functional theory (DFT) calculations, as shown in Figure 2. The N_EF_ descriptor successfully predicted the enhancement of CO_2_ methanation performance over the Ni_3_Fe catalyst, due to a favourable change in the electronic properties of the active Ni phase, while Ni_3_Cu alloy formation was detrimental for the production of CH_4_. In a follow-up study, Ray et al. [45] also included Ni_3_Co catalysts in their calculations and concluded that Co alloying could also enhance the CO_2_ methanation performance but by a smaller degree compared to Fe. They also showed that Co alloying led to the highest promotion regarding the dry methane reforming (DMR) reaction, with the location of the Ni d-band centre (ε_d_) being the most appropriate descriptor to assess the catalytic activity for this reaction.

The Grunwaldt group have been amongst the pioneers in the development of NiFe-based methanation catalysts and the elucidation of the role of Fe in the overall catalytic performance. Mutz et al. [26] prepared Ni_3_Fe catalysts supported on Al_2_O_3_ via deposition–precipitation. The formed alloy nanoparticles exhibited a small size and high dispersion, and the NiFe-based catalyst proved to be more active and stable at lower temperatures compared to the monometallic Ni-based catalyst. The alloy catalyst was also proven to have a quite stable and robust performance after a 45 h time-on-stream operation under industrially oriented conditions [26]. No carbon deposition could be observed under various gas feeds for such catalysts using operando Raman spectroscopy [46]. Farsi et al. [47] investigated the CO_2_ methanation kinetics on such Ni_3_Fe methanation catalysts under technical operation conditions. CO selectivity over CH_4_ was found to increase over shorter residence times and higher temperatures, while water concentration was indicated as the main inhibiting factor.

**Figure 3:**
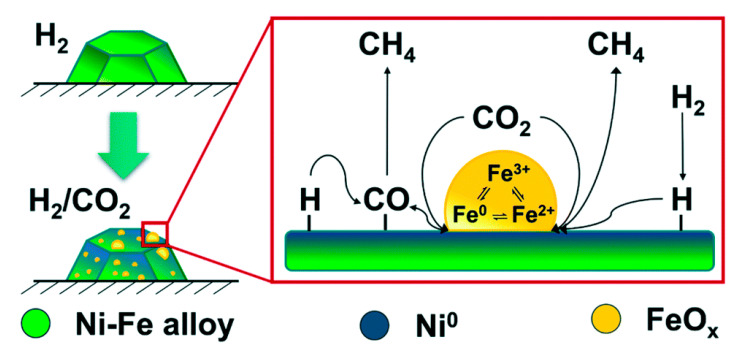
Scheme of the CO_2_ activation mechanism on Ni–Fe alloy-based catalysts during methanation reaction under realistic conditions. Reproduced with permission from [49]. Copyright: Royal Society of Chemistry, London, UK, 2020.

Furthermore, Serrer et al. [48] focussed specifically on the role of Fe during CO_2_ methanation, employing advanced operando spectroscopic methods. Fe was shown to act as a protective or “sacrificial” dopant upon cases of H_2_-dropout during CO_2_ methanation. While Ni-based catalysts are oxidized under such events and then fail to regain their initial activity upon the restoration of H_2_ flow, NiFe-based catalysts retain the Ni^0^ sites in their reduced state, due to the preferential oxidation of their Fe sites into FeO. Under normal operation, Fe was shown to increase the reducibility of Ni and result in the formation of small FeO_x_ clusters at the surface of the alloy nanoparticles, with Fe being found in various oxidation states [49]. It has been suggested that increased methanation performance of NiFe alloy catalysts could be due to these small FeO_x_ clusters on top of alloy nanoparticles that can potentially favour CO_2_ chemisorption and activation, as shown in Figure 3.

**Figure 4:**
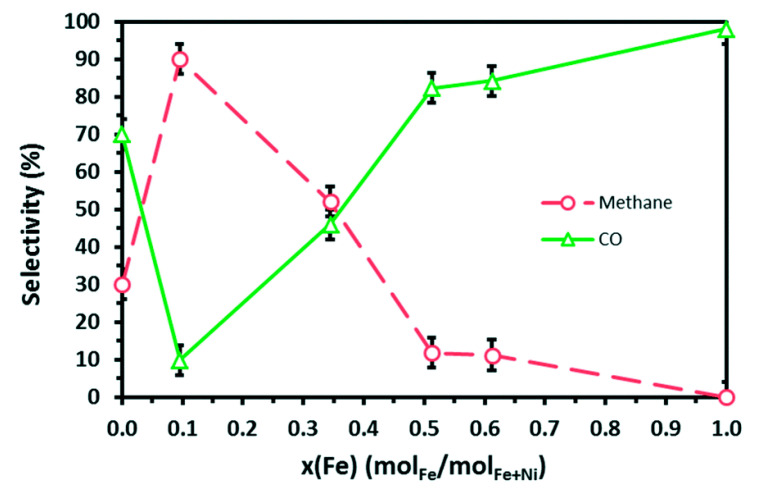
Effect of Fe content on CH_4_ (○) and CO (△) selectivity in comparison to sole metals. The highest methane selectivity, with 90%, was achieved for the catalyst containing 10% Fe. Reproduced with permission from [39]. Copyright: Royal Society of Chemistry, London, UK, 2018.

Mebrahtu et al. [39] used hydrotalcite-precursors with a tailored Fe/Ni ratio in order to prepare NiFe/(Mg,Al)O_x_ catalysts with high levels of metal intermixing and dispersion. The Fe/Ni ratio played a crucial role in the physicochemical properties and the catalytic performance of the prepared catalysts. An Fe/(Ni + Fe) ratio of 0.1 was shown to provide high metal dispersion, small nanoparticle sizes and an optimum amount of surface basic sites. Consequently, the catalyst with this specific ratio offered the best low-temperature catalytic performance in terms of CO_2_ conversion and CH_4_ selectivity, whereas catalysts with higher Fe contents experienced a significant drop for these values (Figure 4). Mebrahtu et al. [50] also indicated a possible deactivation pathway for monometallic Ni catalysts via the formation of Ni hydroxides caused by the water produced in situ upon methanation. It was found that the introduction of Fe prevented the formation of Ni-OH species, thus increasing the catalytic activity of such systems. It was also argued that Fe formed spinel phases on the alumina nanosheets and did not form alloys with Ni.

Giorgianni et al. [51] attributed the beneficial role of Fe into such hydrotalcite-derived catalysts to the presence of Fe(II) species. These species could activate CO_2_ molecules and adsorb H_2_O produced in situ during the reaction, thus preventing the hydroxylation of nearby Ni^0^ active sites. However, these Fe(II) species inevitably undergo oxidation into Fe(III) over time, reducing the catalytic performance.

In a similar fashion, Huynh et al. [52] observed high CO_2_ methanation activity and stability for cheap hydrotalcite-derived NiFe/(Mg,Al)O_x_ catalysts, which could achieve around 75% CO_2_ conversion and 95% CH_4_ selectivity at just 300 °C under high gas space velocities. They also showed that an Fe/(Ni + Fe) ratio of 0.2 (Ni_4_Fe) could lead to the highest promotion in CH_4_ yield at low temperatures, due to a considerable decrease in the activation energy for CH_4_ formation [53]. The formate pathway was observed to be favoured over the direct dissociation of CO_2_ into CO via in-situ diffuse reflectance infrared Fourier transform spectroscopy (DRIFTS) and density functional theory (DFT) [53]. Furthermore, NiFe-containing layered double hydroxides (LDHs) could be in situ grown over alumina and silica washcoated cordierite monoliths via an urea hydrolysis preparation method [54]. Using different washcoat materials and metal concentrations, a structured NiFe bimetallic catalyst with a thin catalytic layer on the cordierite substrate was prepared. This structured NiFe monolithic catalyst achieved high CH_4_ yields under high gas flow rates, thus making it attractive for industrial-scale applications.

Burger et al. [55] prepared NiAlO_x_ coprecipitated catalysts modified with Fe and Mn. Both metals improved the performance of the NiAlO_x_ catalysts. Mn formed separate MnO_x_ phases that enhanced the CO_2_ adsorption capacity and metal dispersion, while Fe formed NiFe alloys, promoting the electronic properties of Ni and enhancing the catalyst stability. The optimal Ni to promoter molar ratio was five. Such catalysts were also proven to be resistant upon sulphur poisoning, since H_2_S was preferentially adsorbed at the metal promoter phases [56], thus preserving the Ni active sites [57].

Burger et al. [58,59] also studied the mechanism of Fe promotion in coprecipitated Ni/Al_2_O_3_ catalysts. In one study [58], Fe was deposited adjacent to Ni nanoparticles via a surface redox mechanism creating alloyed surface phases. Fe alloying also increased the activity of Ni/Al_2_O_3_ catalysts prepared via deposition–precipitation and the thermal stability of co-precipitated NiAlO_x_ catalysts. (γFe,Ni) alloy nanoparticles were observed at co-precipitated NiFeAlO_x_ catalysts and the presence of Fe(II) species upon aging provided additional CO_2_ activation sites [59]. The generation of active Fe(II) species appeared to partially offset the negative effects caused by active metal sintering upon aging.

Other reports about Fe promotion in Ni/Al_2_O_3_ catalysts include the work of Li et al. [60]. It was found that adding 3% Fe on a 12% Ni/Al_2_O_3_ catalyst led to a mild improvement for CO_2_ conversion and CH_4_ selectivity, whereas the increase in the Fe content to 12% (Fe/Ni ≈ 1) caused a worse methanation performance. Liang et al. [61] studied the effect of various additives on Ni/Al_2_O_3_ catalysts and found the presence of an increased number of oxygen vacancies over the Fe-modified catalyst, as evidenced by electron paramagnetic resonance (EPR), that caused favourable changes to the reaction pathway. Finally, in contrast to other works, Daroughegi et al. [62] found that a 25% Ni/Al_2_O_3_ catalyst modified with 5% Fe exhibited a much worse methanation performance in terms of CO_2_ conversion compared to the corresponding monometallic Ni catalyst.

Up until now, the NiFe-based catalysts studied were supported on Al_2_O_3_ or Al-based “inert” supports. The oxide support can, however, also play a major role in the methanation performance of alloy catalysts [63]. Ren et al. [64] showed that modification of a 30% Ni/ZrO_2_ catalyst with 3% Fe enhanced its low temperature methanation performance. Modification with 5% Fe also yielded better results compared to the monometallic catalyst, but higher Fe contents led to a decline in methanation activity. The authors suggested that the majority of Fe in the catalysts was not fully reduced following pretreatment under H_2_ flow, but remained at an Fe(II) oxidation state. These species could potentially improve the dispersion and reducibility of the Ni phase and also promote the reduction of ZrO_2_, thus facilitating the presence of oxygen vacancies, that together with Fe(II) sites typically enhance CO_2_ chemisorption and dissociation.

**Figure 5:**
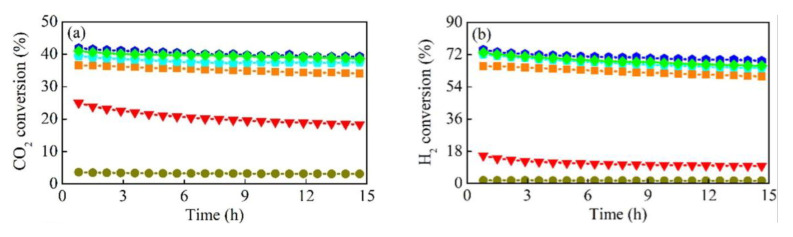

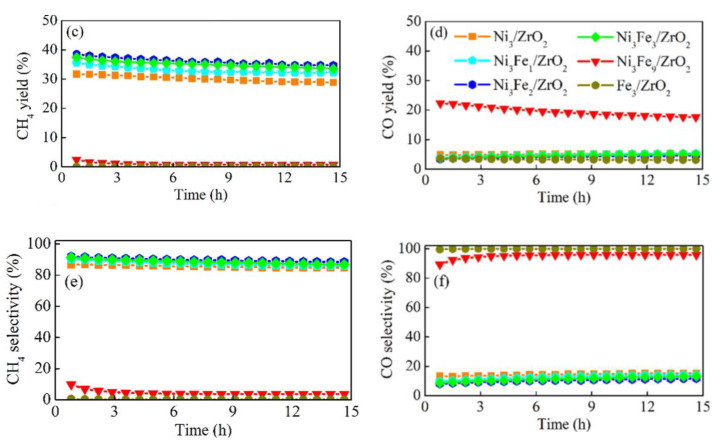
Conversions of (**a**) CO_2_ and (**b**) H_2_, yields of (**c**) CH_4_ and (**d**) CO, and selectivities of (**e**) CH_4_ and (**f**) CO on ZrO_2_-supported catalysts plotted versus time on stream for reaction of CO_2_ and H_2_ (5 mL/min CO_2_ + 10 mL/min H_2_ + 25 mL/min Ar) at 673 K. Reproduced with permission from [65]. Copyright: Elsevier, Amsterdam, The Netherlands, 2019.

Yan et al. [65] employed low metal loadings of Ni and Fe (1.5% Ni and 0.5–4.5% Fe) supported ZrO_2_ and assigned the various interfacial sites on the catalysts as selective for different CO_2_ hydrogenation products. The Ni-ZrO_2_ interface in the monometallic catalyst was characterized as active for the methanation reaction. Fe addition up to an equimolar amount to that of Ni led to a small improvement in CO_2_ conversion and CH_4_ selectivity and probably preserved the methane selective active metal–ZrO_2_ interface. Only with the addition of a large amount of Fe (Fe/Ni ratio at 3) does the CO selectivity increase, via the creation of Ni-FeO_x_ interface that binds intermediate CO weakly and favours the reverse water gas shift (RWGS) reaction. Their experimental results are summarized in Figure 5. Furthermore, other studies showed that Fe promotion provided an enhancement of the methanation performance of Ni-based catalysts supported on Al_2_O_3_ and mesoporous clay modified with ZrO_2_ [66,67].

It has been previously mentioned that CeO_2_ support can offer significant advantages regarding the CO_2_ methanation performance of Ni-based catalysts, when compared with other metal oxide supports [12]. However, Fe modification of Ni catalysts supported on CeO_2_-based supports appears to exert a negative influence on CO_2_ methanation. Winter et al. [68] studied CeO_2_-supported NiFe catalysts with low metal loadings of Ni and Fe (1.5% Ni and 0.5–1.5% Fe). In contrast to the ZrO_2_ supported catalysts reported by Yan et al. [65], Fe modification, even in small amounts, led to a significant drop in CO_2_ conversion and rise in CO selectivity. The majority of Fe remained oxidized according to X-ray absorption near edge structure (XANES) analysis and these oxidized Fe species probably led to a weakened interaction between metal and intermediate CO, thus facilitating CO desorption and higher CO selectivity [68].

Pastor-Pérez et al. [69] prepared Fe and Co modified Ni catalysts supported on CeO_2_-ZrO_2_ and observed a negative effect of Fe addition, since the 3% Fe, 15% Ni/CeO_2_-ZrO_2_ catalyst exhibited lower values for CO_2_ conversion and CH_4_ selectivity compared to the monometallic Ni catalyst. Likewise, le Saché et al. [70] observed that Fe addition on a Ni/CeO_2_-ZrO_2_ catalyst led to a drop in CO_2_ conversion and CH_4_ selectivity. Furthermore, Frontera et al. [71] also reported a drop in CO_2_ methanation activity upon Fe alloying over Ni catalysts supported on Gd-doped CeO_2_ (GDC). In contrast to NiFe catalysts supported on inert supports such as Al_2_O_3_ and SiO_2_ [26,43], Fe addition appeared to suppress the population of surface oxygen vacancies on the already defect-rich GDC support.

**Figure 6:**
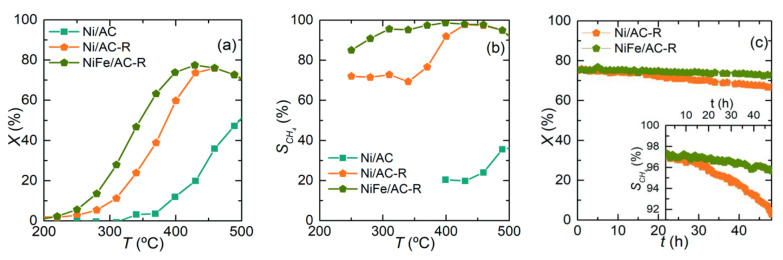
Comparison of the catalytic properties of the best Ni catalyst Ni/AC–R and the same promoted by Fe NiFe/AC–R: X_CO2_ and S_CH4_ as a function of temperature (**a**,**b**) as well as time-on-stream (TOS) at 450 °C (**c**). Reaction conditions: P = 1 bar; Weight Hourly Space Velocity (WHSV) = 60,000 mL g^–1^ h^–1^; CO_2_:H_2_ = 1:4. Reproduced with permission from [73]. Copyright: Royal Society of Chemistry, London, UK, 2020.

Carbon is another type of support in heterogeneous catalysis, which has been deemed as inactive regarding CO_2_ methanation when using Ni-based catalysts [72]. However, Gonçalves et al. [73] managed to improve the performance of activated carbon (AC) supported Ni catalysts by modifying the surface chemistry of carbon and by introducing Fe as a second metal. They reported that 5% Fe addition on a 15% Ni catalyst supported on activated carbon with increased basicity (AC-R) improved the catalytic performance for CO_2_ methanation. More specifically, NiFe alloy formation improved the low-temperature activity of the supported catalysts, increased the CH_4_ selectivity and favoured catalyst stability, as shown in Figure 6.

The formation of NiFe alloy phase can sometimes take place via the migration of Fe atoms that are incorporated in the support lattice, towards the surface Ni particles, upon exposure to a reducing atmosphere. Wang et al. [74] impregnated Ni on an olivine support and observed the formation of NiFe alloyed phase from the Fe contained in the support. The good CO_2_ methanation performance was ascribed to NiFe alloy formation and its favourable interaction with unreduced FeO_x_ segregated from the olivine support upon calcination and reduction treatments. Thalinger et al. [75], however, reported that NiFe alloy formation through Fe exsolution from a reducible La_0.6_Sr_0.4_FeO_3-δ_ perovskite with supported Ni nanoparticles could spoil the methanation chemistry of Ni through an unfavourable change in nickel’s electronic properties. The suppression of CO_2_ methanation was less pronounced on a Ni/SrTi_0.7_Fe_0.3_O_3-δ_ catalyst, due to the better structural stability of this perovskite support [76] and, thus, the lesser extent of Fe exsolution and alloying with Ni.

Lastly, Pandey et al. [77] performed a study in which they used unsupported NiO as a catalyst precursor and introduced various Fe amounts. Similarly to many of their supported counterparts [26,39], Ni catalysts modified with Fe in a stoichiometry of Ni/Fe > 1 exhibited an increase in catalytic activity for CO_2_ methanation compared to metallic Ni catalyst. The catalysts that consisted of Ni-rich NiFe alloys with 10% Fe and 25% Fe yielded the best results. The one with 10% Fe was shown to be the most active and the higher activity was ascribed to the absence of unalloyed Fe in this catalyst [77].

By taking into consideration the large number of literature reports regarding Fe modified Ni catalysts [26,39,65], it can be concluded that, under specific circumstances, Fe can significantly promote the CO_2_ methanation performance. The molar ratio between Ni and Fe appears to be the most important factor that determines whether Fe will promote or demote the methanation performance. An Fe/Ni ratio between 0.1 and 0.25 mostly yields the best results, while Fe-rich alloys significantly degrade the catalytic activity [39]. Furthermore, the type of support also influences the degree of promotion. Al_2_O_3_, ZrO_2_, SiO_2_ and carbon supported Ni catalysts are typically promoted upon Fe addition at a specific Fe/Ni ratio, but Ni catalysts supported on defect-rich CeO_2_-based supports do not exhibit a similar behaviour [42,68]. Through spectroscopic techniques, it has been reported that partly reduced Fe(II) species play a major role in the promotion mechanism [49,50]. In general, Fe constitutes an ideal promoter for Ni-based catalysts, since it is also a cheap and earth-abundant metal and, through the favourable formation of NiFe alloy, it can be used to explicitly tailor the electronic properties of the catalytically active phase. Some of the most representative studies that include Ni-M (M = transition metal) bimetallic catalysts for CO_2_ methanation are comparatively presented in Table 1.

### 2.2. Promotion with Co

Co is another transition metal commonly used to prepare bimetallic NiCo catalysts for CO_2_ methanation. Co is right adjacent to Ni in the periodic table, it can dissolve into the lattice of metallic Ni similarly to Fe and its easy transition between the oxidation states of Co(III), Co(II) and Co^0^ can induce modifications to the electronic properties of Ni-based catalysts [24,78]. Among the first works, Guo et al. [79] prepared several bimetallic NiCo catalysts supported on SiO_2_ with different Co/Ni ratios. They found that higher Co loadings improved the activity of the catalysts and that a Co/Ni ratio of 0.4 led to the best performing catalyst. The formation of a homogeneous NiCo alloy supported on SiO_2_ has also been shown to promote CO dissociation and hydrogen spillover during CO methanation, leading to higher activity [78].

**Figure 7:**
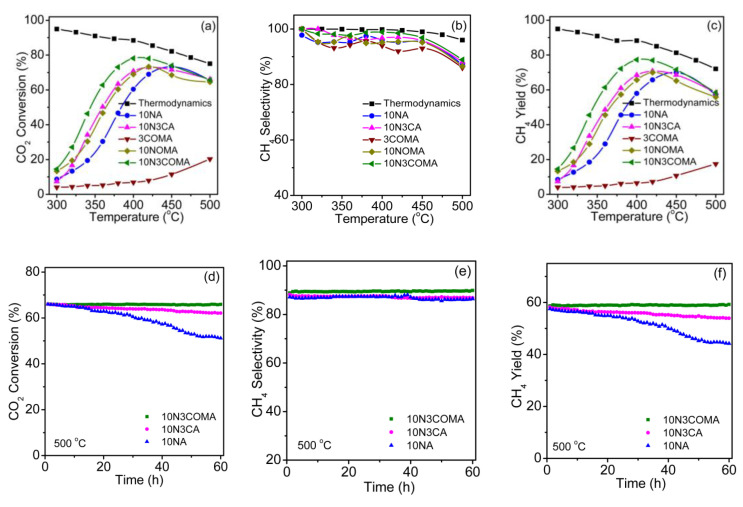
Catalytic properties of the catalysts: (**a**,**d**) CO_2_ conversion, (**b**,**e**) CH_4_ selectivity, and (**c**,**f**) CH_4_ yield. Reproduced with permission from [37]. Copyright: Elsevier, Amsterdam, The Netherlands, 2018.

Further works focussed on Al_2_O_3_ supported NiCo catalysts, with Xu et al. [80] and Liu et al. [37] introducing ordered mesoporosity into the catalyst structure via a one-pot evaporation induced self-assembly (EISA) synthesis method. Xu et al. [80] reported that a suitable Co/(Ni + Co) ratio of 20% could increase the catalytic activity and stability. The authors suggested that Ni and Co were supported as adjacent monometallic phases on the mesoporous Al_2_O_3_ structure and that they served as active sites for the chemisorption of H_2_ and CO_2_, respectively. The synergy between these two metals acted to decrease the activation energy for CO_2_ methanation. Liu et al. [37] also prepared similar ordered mesoporous NiCo/Al_2_O_3_ composites. The authors claimed that NiCo alloy formation and the confinement effect of the mesoporous structure were responsible for the improved low-temperature activity and stability of the bimetallic catalysts. Their best performing 10N3COMA catalyst (3% Co_3_O_4_ and 10% NiO on ordered mesoporous Al_2_O_3_) exhibited 78% CO_2_ conversion and 99% CH_4_ selectivity at 400 °C, as well as great stability upon a 60 h time on stream test, as shown in Figure 7.

Alrafei et al. [81] prepared Ni and NiCo catalysts with various metal loadings supported on Al_2_O_3_ extrudates. According to their findings, 10% Ni and 10% Co were the most suitable metal loadings for the bimetallic catalysts. The bimetallic catalyst with these metal loadings outperformed the monometallic 10% Ni catalyst, as Co helped to increase the reducibility and dispersion of the Ni phase. However, the 20% monometallic Ni catalyst exhibited better performance in terms of CO_2_ conversion and CH_4_ selectivity when compared to the bimetallic catalyst, with a total Ni and Co metal loading of 20%. All catalysts presented a remarkable stability upon 200 h of operation. In contrast to other works, Fatah et al. [82] reported that 5% Co addition on a 5% Ni/Al_2_O_3_ catalyst caused roughly a 30% drop in CO_2_ conversion and lower CH_4_ selectivity compared to the Ni monometallic catalyst. The deactivation was attributed to the occurrence of larger particles in the NiCo bimetallic catalyst, while an increased formation of formate intermediate species was also identified via in situ FTIR.

Besides SiO_2_ and Al_2_O_3_, a lot of works focus on NiCo catalysts supported on ZrO_2_, CeO_2_-ZrO_2_ and other CeO_2_-based supports. As discussed before, Ren et al. [64] studied 30% Ni/ZrO_2_ catalysts modified with Fe, Co and Cu. The Co-modified catalyst exhibited higher CO_2_ conversion, but slightly lower CH_4_ selectivity, and Fe was shown to be more suitable as a promoter metal. Razzaq et al. [83], on the other hand, concluded that a Co promoted Ni/CeO_2_-ZrO_2_ catalyst was more suitable compared to other catalysts (modified with Mo and Fe) for the co-methanation of CO and CO_2_ in CH_4_-rich coke oven gas. Following that work, Zhu et al. [84] confirmed that 5% Co addition on a 15% Ni/CeO_2_-ZrO_2_ catalyst could promote catalytic activity and stability for CO_2_ methanation. The optimum CeO_2_/ZrO_2_ molar ratio of 1/3 (Ce_0.25_Zr_0.75_O_2_) was shown to also play a major role by providing a maximum amount of oxygen vacancies close to active metal sites that favour CO_2_ activation and dissociation.

As previously discussed, Pastor-Pérez et al. [69] prepared Fe- and Co-modified Ni catalysts supported on CeO_2_-ZrO_2_. While Fe modification impeded the CO_2_ methanation activity, the addition of 3% Co on a 15% Ni/CeO_2_-ZrO_2_ catalyst enhanced the CO_2_ conversion, CH_4_ selectivity and the long-term stability and it allowed for the use of higher space velocities. Following that work, Pastor-Pérez et al. [85] also showed that such Co modified Ni catalyst could prevent coke deposition during the reaction. The catalyst was quite robust upon long-term operation tests, while the presence of methane in the feed gas did not appear to impede the catalytic activity, but instead promoted it. In contrast to other works, the addition of Co in Ni catalysts supported on defect-rich GDC support did not offer any advantages to the catalytic performance for CO_2_ methanation, as reported by Frontera et al. [86].

Apart from the typical catalyst supports, Jia et al. [87] supported the two metals of Ni and Co on TiO_2_ coated silica spheres. The electronic effect of the reducible TiO_2_ layer acted to promote metal dispersion and the adsorption of H_2_ and CO_2_, while the addition of Co second metal enhanced the intrinsic activity and long-term stability of the NiCo/TiO_2_@SiO_2_ catalyst in a fluidized bed reactor. Varun et al. [88] prepared NiO-MgO nanocomposites, via a sonochemical method, that were active for the CO_2_ methanation reaction. The authors further modified the composites by impregnating 2% Co, Fe and Cu. Among the three dopants, only Co led to significant activity improvement by lowering the activation energy for the methanation pathway.

**Figure 8:**
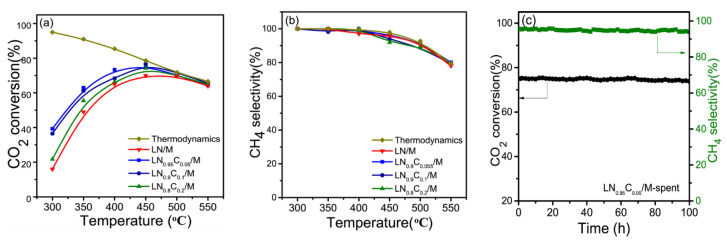
Catalytic properties of the catalysts at 60,000 mL g^−1^ h^−1^: (**a**) CO_2_ conversion, (**b**) CH_4_ selectivity, and (**c**) lifetime test of LN_0.95_C_0.05_/M at 450 °C. Reproduced with permission from [89]. Copyright: Elsevier, Amsterdam, The Netherlands, 2020.

Lastly, Zhang et al. [89] attempted to increase the metal intermixing between Ni and Co by incorporating Co into the lattice of LaNiO_3_ perovskite and supporting the perovskite on a mesostructured cellular foam (MCF) silica support. Following reduction, NiCo alloy nanoparticles and adjacent La_2_O_3_ phase, highly dispersed on the MCF support, were generated. The promoting effect of Co alloying, the increased basicity and dispersion induced by the La_2_O_3_ phase and the confinement effect of the mesoporous support acted to yield a catalyst with increased activity and great stability. A low Co/(Ni + Co) ratio of 5% at a perovskite with nominal composition LaNi_0.95_Co_0.05_O_3_ was found to be optimal by leading to NiCo alloy particles with the smallest size (Figure 8).

Overall, it can be concluded that Co mostly acts to promote the performance of CO_2_ methanation catalysts. However, based on the reported literature, it appears that the type of support, either inert or reducible, does not affect the promotion mechanism [37,69]. The effect of the Co/(Ni + Co) ratio is also not as apparent when compared to similar NiFe bimetallic catalysts. However, it appears that Ni-rich NiCo catalysts lead to a more favourable activity [89].

### 2.3. Promotion with Cu

In all of the works discussed until now, whenever Cu is added in Ni-based catalysts, the CO_2_ methanation reaction is hindered and the antagonistic reverse water–gas shift (RWGS) reaction is instead promoted. Thus, the Cu-containing bimetallic catalysts turned out to be inferior to monometallic Ni ones for CO_2_ methanation [38,45,64,88]. Dias et al. [90] also showed that even 1% Cu addition on 10% Ni/SiO_2_ could drastically decrease CO_2_ conversion and increase the selectivity for CO. Although Cu enhanced the dispersion and reducibility of Ni, similarly to other transition metals (Fe and Co), it deactivated the catalyst for the CO_2_ methanation reaction. This was probably a result of NiCu alloy formation and the inability of pure Cu and NiCu alloy to adsorb hydrogen. Despite that, the Cu-containing catalysts could be suitable for CO production, due to their high stability and CO selectivity. Table 1 below summarises typical bimetallic Ni-based catalysts promoted with transition metals (Fe, Co and Cu) for the methanation of CO_2_.

## 3. Promotion with Noble Metals

Although noble metals are significantly pricier than transition metals, they can provide significant advantages regarding the CO_2_ methanation reaction, due to their excellent low-temperature activity, as well as their high reducibility and stability once oxidized [18,19,91,92,93]. The combination of Ni with a noble metal aims to transfer some of these properties into Ni-based catalysts, without the need for the use of a high noble metal loading [33]. Combined Ni and Ru catalysts exist mostly in the form of heterostructures, rather than alloys, and Ru can provide additional methanation sites, as well as spillover hydrogen into nearby Ni sites [94]. On the other hand, Pt and Pd mostly modify the electronic properties of Ni via alloy formation [31,32].

### 3.1. Promotion with Ru

Ru is the cheapest among the discussed noble metals and, at the same time, the most active metallic phase in CO_2_ methanation. The combination of the metals Ru and Ni has been described as “privileged” due to the great benefits that can arise from the NiRu bimetallic synergy [94]. Ru can drastically improve the reducibility of Ni catalysts, as well as improve the Ni metallic dispersion and provide additional methanation sites.

**Figure 9:**
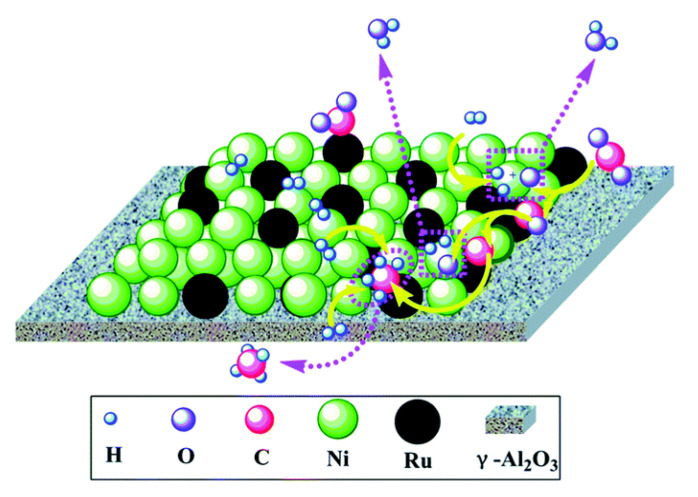
The proposed a possible reaction mechanism of CO_2_ methanation over 10Ni–1.0Ru catalyst. Reproduced with permission from [30]. Copyright: Royal Society of Chemistry, London, UK, 2014.

In an early work, Zhen et al. [30] impregnated Ni and Ru on γ-Al_2_O_3_. Ru was found to be segregated at the outer surface and not forming an alloy with the dominant Ni metallic phase. The catalyst with 1% Ru and 10% Ni presented high activity, CH_4_ selectivity and stable performance upon 100 h of operation. It was suggested that CO_2_ molecules dissociated on Ru particles and H_2_ molecules on Ni ones, followed by hydrogen spillover to hydrogenate the adsorbed carbon species (Figure 9). Lange et al. [95] also found that part of Ru noble metal could be substituted by Ni in Ru/ZrO_2_ catalysts without compromising the catalytic activity. They claimed that low metal loadings led to the formation of alloys, but higher loadings led to the separation of the two metallic phases.

Hwang et al. [96] modified the Ru content on a 35% Ni and 5% Fe/Al_2_O_3_ xerogel. A volcano-shaped trend was observed regarding the Ru content, with the small loading of 0.6% yielding the best results. Liu et al. [97] synthesized Ni and Ru-doped ordered mesoporous CaO-Al_2_O_3_ nanocomposites. The confinement of the active nanoparticles due to the ordered mesoporous structure of the catalyst prevented their sintering, while the CaO component increased the basicity and favoured CO_2_ chemisorption. The addition of a small quantity of Ru as a second metal enhanced the activation of H_2_ and CO_2_, due to its synergy with the Ni primary phase, leading to a final nanocomposite catalyst with superior catalytic activity and stability. In a more recent study, Chein et al. [98] also reported that a 1% Ru and 10% Ni/Al_2_O_3_ catalyst had a better low-temperature activity compared to the respective monometallic catalysts, also agreeing with the fact that a small amount of added Ru can induce significant changes to the catalytic activity of Ni-based catalysts.

NiRu bimetallic catalysts are also viable candidates to be studied under more industrial-like conditions. Bustinza et al. [99] prepared bimetallic NiRu structured monolithic catalysts with low Ru contents by homogeneously dispersing Ni and Ru precursors over alumina washcoated monoliths. Through an appropriate preparation method, Ni was supported in the form of small nanoparticles (2–4 nm), while Ru was atomically dispersed over the structured support. The bimetallic catalyst with these characteristics provided stable CO_2_ methanation performance for many hours under high space velocities. Navarro et al. [100] structured a catalyst consisting of MgAl_2_O_4_ supported 0.5% Ru and 15% Ni washcoated on metal micromonoliths. The structured catalyst was stable upon 100 h of continuous operation. The effect of CH_4_ presence in the initial gas stream (simulated biogas) was also addressed by Stangeland et al. [101]. CO_2_ conversion was increased upon promotion of a 20% Ni/Al_2_O_3_ catalyst with 0.5% Ru and the catalyst was not greatly affected by the CH_4_ presence in the gas stream, exhibiting a stable methanation activity.

Another practical implementation of the NiRu bimetallic combination is in dual-function materials (DFMs), used for the integrated capture and methanation of CO_2_ [102]. Having Ni active methanation phase in DFMs presents a major problem, since Ni is deactivated during the CO_2_ capture step from flue gases and cannot be reactivated under H_2_ flow at the usual operation temperature of 320 °C [21,103]. Adding just 1% Ru in a DFM consisting of 10% Ni increased the reducibility of the Ni phase by 70% and thus enabled CH_4_ production under isothermal cyclic operation [104]. Employing in-situ DRIFTS, it was shown that both Ru and Ni are active sites for the methanation reaction, with bicarbonate and bidentate carbonate intermediate species spilling over to both metals before being hydrogenated into CH_4_ [105].

Besides Al_2_O_3_-based supports, ZrO_2_, CeO_2_-ZrO_2_ and other CeO_2_-based defect-rich supports are also commonly used in NiRu-based catalysts and will be discussed thereafter. Ocampo et al. [106] modified 5% Ni catalysts supported on CeO_2_-ZrO_2_ by changing the CeO_2_/ZrO_2_ ratio and by adding a noble metal phase (Ru and Rh). A mass ratio between CeO_2_ and ZrO_2_ of 1.5 in the support led to the best results and 0.5% noble metal addition increased the catalytic activity and stability by enhancing Ni dispersion. In a later study, Shang et al. [107] prepared Ru-doped 30% Ni catalysts supported on CeO_2_-ZrO_2_ via an one-pot hydrolysis method. They found that Ru addition could enhance Ni dispersion and promote the basicity of the catalysts, thereby achieving a quite increased low-temperature activity. They reported that their best performing catalyst 3% Ru, 30% Ni/Ce_0.9_Zr_0.1_O_2_ could achieve 98.2% CO_2_ conversion and 100% CH_4_ selectivity under the reaction conditions tested.

**Figure 10:**
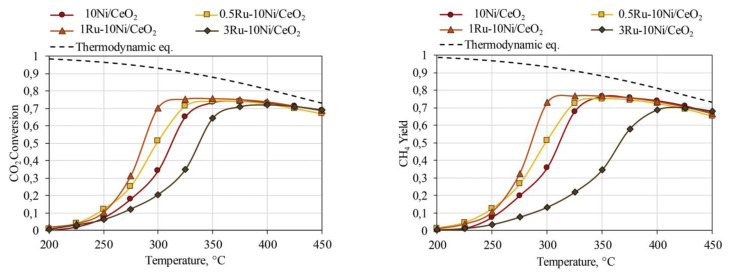
CO_2_ conversion and CH_4_ yield trends for the samples doped with 0.5%, 1% and 3% ruthenium (H_2_:Ar:CO_2_ = 4:5:1; WHSV = 60 l/(h·g_cat_)). Reproduced with permission from [33]. Copyright: Elsevier, Amsterdam, The Netherlands, 2020.

Renda et al. [33] performed an extensive study on the effect of noble metal loading on the catalytic activity of 10% Ni catalysts supported on CeO_2_. At first, CeO_2_ was identified as the most suitable catalyst support over CeZrO_4_ and CeO_2_/SiO_2_, which is in agreement with other studies [12]. In the case of Ru, a volcano-shaped trend could be observed between the Ru loading and the yield for methane at low-temperatures, with 1% Ru leading to the best performance, as shown in Figure 10. The yield for CH_4_ at 300 ^o^C was almost double for the 1% Ru promoted 10% Ni/CeO_2_ catalyst since Ru offered additional active sites for CO_2_ methanation in synergy with Ni. The worse performance at higher Ru loadings was attributed to the worsening of active metal dispersion. Renda et al. [108] also showed that using ruthenium acetylacetonate instead of ruthenium chloride as the Ru precursor salt could improve the dispersion of the active metals, due to the templating effect of the precursor salt, leading to an enhanced methanation performance.

**Figure 11:**
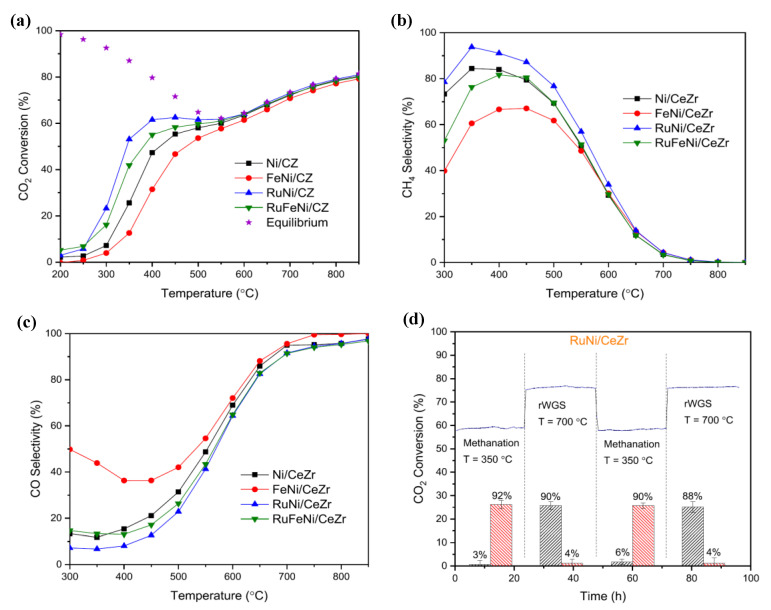
(**a**) CO_2_ conversion, (**b**) CH_4_ selectivity and (**c**) CO selectivity for all catalysts as a function of temperature. (**d**) Stability test for the RuNi/CeZr catalyst, varying the temperature between 350 and 700 °C and starting with the methanation cycle. Product selectivity is represented as columns, black for CO and red for CH_4_. Reproduced with permission from [70]. Copyright: American Society of Chemistry, Washington, DC, USA, 2020.

In another interesting study, le Saché et al. [70] reported that Ru addition on a Ni/CeO_2_-ZrO_2_ catalyst could improve the dispersion of Ni and increase the overall intrinsic activity for the reduction of CO_2_. The bimetallic NiRu catalyst exhibited enhanced CO_2_ methanation performance in terms of CO_2_ conversion and CH_4_ selectivity at lower temperatures compared to the monometallic Ni catalyst. Furthermore, the NiRu-based catalyst was also active for CO production via the reverse water–gas shift (RWGS) reaction upon temperature increase. It was shown that the catalyst could be stable and high-performing over long time for both reactions (CO_2_ methanation and RWGS) at the respective reaction temperatures (350 and 700 °C). Their experimental results are summarized in Figure 11. It should also be noted that Fe addition on the Ni/CeO_2_-ZrO_2_ catalyst led to inferior CO_2_ methanation performance, as discussed previously.

Through a novel preparation method, Polanski et al. [109] prepared Ru nanoparticles supported on metallic Ni grains via first preparing Ru nanoparticles supported on an intermediate silica carrier. Ru and Ni were then deposited on the carrier and the silica was etched upon suspension in a strong basic solution to yield a 1.5% Ru/Ni catalyst with an oxide passivation layer. The prepared catalyst exhibited a greatly enhanced low-temperature activity with about 100% CO_2_ conversion being achieved at a temperature as low as 200 °C. Carbon deposition was shown to deactivate the catalyst over time, with the initial activity being regained upon treatment under H_2_ atmosphere. In a follow-up work, Siudyga et al. [110] synthesized 1.5% Ru catalysts supported on Ni nanowires via a similar preparation method. The higher surface area of the 1D nanostructure of the Ni support provided an additional advantage, with around 100% CO_2_ conversion being reached at 179 °C and the onset temperature for the reaction being observed at just 130 °C. Siudyga et al. [111] also observed that Ru/Ni catalysts are highly active for CO methanation from syngas, with 100% CO conversion being reached at 178 °C and observable CO conversion being detected even at −7 °C.

The choice of Ru as a promoter for the CO_2_ methanation reaction in Ni-based catalysts is a sensible option, since the combination of these two metals appears to yield favourable results, as reported by numerous works [30,33,70]. The synergistic effect through the presence of Ru and Ni adjacent phases can be observed via the increased metal dispersion, the higher reducibility, the improved low-temperature activity and the superior stability. Ni and Ru are both very active CO_2_ methanation sites on their own and their combination can lead to an enhancement of the intrinsic catalytic activity, and to a decrease in the activation energy, through the favourable activation of H_2_ and CO_2_ reactant molecules [33,94]. The only drawback for the use of Ru is its higher price compared to transition metals like Ni, Fe and Co, even though it is significantly cheaper when compared to other noble metals such as Rh or Pt. On rare occasions, Ru addition does lead to a drop in CO_2_ methanation activity, like in the work of Wei et al. [112] regarding Ni catalysts supported on zeolites. Some of the most representative studies that include Ni-M (M = noble metal) bimetallic catalysts for CO_2_ methanation are comparatively presented in Table 2.

### 3.2. Promotion with Rh

Rhodium (Rh) is one of the most expensive noble metals and it is known to be highly active for CO_2_ methanation at low temperatures, even when used under very low loadings [19,113]. However, its presence in bimetallic Ni-Rh catalysts does not always lead to a desirable activity increase, especially when taking into consideration the increased cost of the catalytically active material. One of the earlier works that does report a promoting effect of Rh in Ni-based catalysts was conducted by Ocampo et al. [106]. The addition of just 0.5% Rh on a 5% Ni/CeO_2_-ZrO_2_ catalyst greatly increased the catalytic activity of the catalyst, and also by a larger degree compared to the modification with 0.5% Ru. Higher stability for the noble metal modified catalysts was also observed and the better performance was attributed to the enhancement of the dispersion of nickel nanoparticles. Swalus et al. [114] followed a different path by mechanically mixing 1% Rh/Al_2_O_3_ and 1% Ni/activated carbon catalysts. The combined catalyst presented increased methane production through a synergistic collaboration between Ni and Rh metallic sites. More specifically, Ni sites were active for H_2_ adsorption and Rh sites for CO_2_ adsorption, respectively. Active hydrogen species could then spill over to Rh sites to facilitate the hydrogenation of chemisorbed CO_2_ molecules.

Via a complex material architecture, Arandiyan et al. [115] prepared highly active NiRh CO_2_ methanation catalysts. They first synthesized a 3-dimensionally ordered macroporous (3DOM) perovskite with nominal stoichiometry LaAl_0.92_Ni_0.08_O_3_ via a poly(methyl methacrylate) (PMMA) colloidal crystal-templating preparation method. Rh was then introduced via wet impregnation of a Rh precursor salt. Upon a high-temperature reduction treatment, exsolution of Ni nanoparticles occurred, followed by NiRh alloy formation with the surface Rh atoms. The Ni exsolution procedure facilitated a high dispersion of Ni on the entire perovskite surface and a strong metal–support interaction that could otherwise not be achieved by traditional impregnation techniques [116]. The Rh-Ni/3DOM LAO catalyst with highly dispersed NiRh alloy nanoparticles on a macroporous perovskite support exhibited a significantly enhanced CO_2_ methanation activity, when compared to similar monometallic Ni and Rh catalysts [115].

**Figure 12:**
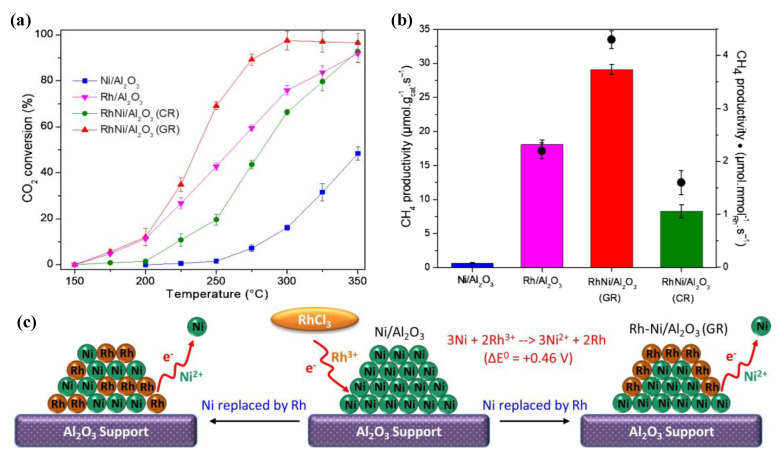
Catalytic activity of Ni/Al_2_O_3_, Rh/Al_2_O_3_, RhNi/Al_2_O_3_ (CR), and RhNi/Al_2_O_3_ (GR) for CO_2_ methanation: (**a**) CO_2_ conversion versus reaction temperature; (**b**) Rh normalised CH_4_ productivity at 250 °C. (**c**) Possible structures of as-prepared bimetallic RhNi/Al_2_O_3_ catalysts synthesised by galvanic replacement. Reproduced with permission from [117]. Copyright: Elsevier, Amsterdam, The Netherlands, 2020.

The architecture of bimetallic NiRh catalysts supported on Al_2_O_3_ was also modified by Wang et al. [117]. On the one hand, the method of galvanic replacement (GR) led to the formation of a Ni@Rh core-shell catalyst, where Ni nanoparticles were encapsulated by an atomically thin RhO_x_ shell. On the other hand, the method of chemical reduction (CR) led to a higher degree of intermixing between Ni and Rh. The catalyst prepared by galvanic replacement presented a superior CO_2_ methanation performance, leading to the conclusion that Rh atoms highly dispersed as a thin shell over Ni nanoparticles were more active compared to large Rh nanoparticles or NiRh alloys (Figure 12). Dissociative adsorption of CO_2_ over Rh was observed as the key intermediate step. Reducing the loading of Rh could also further enhance the specific activity per noble metal atom. Thus, the galvanic replacement method, which rests on the reduction potential difference between the various redox metal pairs, was shown to be an effective strategy for the preparation of bimetallic catalysts [118].

Contrasting the results discussed until now [115,117], there are a number of works that observe a deactivating effect of Rh presence in Ni-based catalysts. Mutz et al. [46] reported that a NiRh_0.1_ catalyst supported on Al_2_O_3_ showed inferior CO_2_ conversion to a similar monometallic Ni catalyst over the entire temperature range tested. Moreover, Mihet et al. [119] and Renda et al. [33] both showed that 0.5% Rh addition on a 10% Ni catalyst supported on Al_2_O_3_ and CeO_2_, respectively, led to a drop in the CO_2_ conversion and the overall CH_4_ yield. A NiRh combined catalyst supported on Al_2_O_3_ and prepared Heyl et al. [120] was also less active for CO_2_ methanation compared to a monometallic Rh catalyst.

Therefore, Rh insertion in Ni-based catalysts does not always lead to an activity enhancement and it appears that certain requirements need to be followed, such as the existence of highly dispersed NiRh alloys with strong metal–support interaction, or the fine dispersion of Rh on top of Ni particles [115,117]. In any case, the very high cost of Rh renders it unattractive for use as promoter in Ni-based catalysts, especially when comparable activity promotion can be achieved via the use of cheaper metals, like Ru.

### 3.3. Promotion with Pt

**Figure 13:**
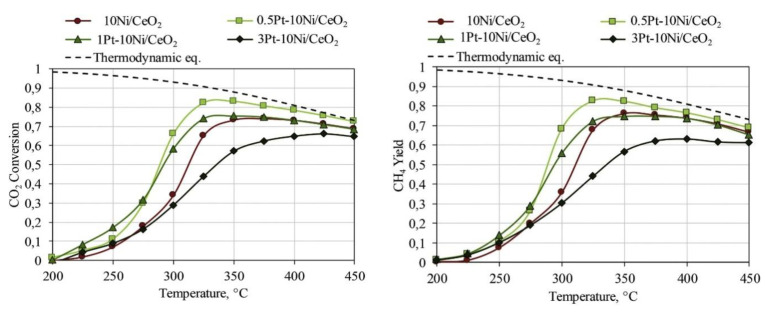
CO_2_ conversion and CH_4_ yield trends for the samples doped with 0.5%, 1% and 3% of platinum (H_2_:Ar:CO_2_ = 4:5:1; WHSV = 60 L/(h·g_cat_)). Reproduced with permission from [33]. Copyright: Elsevier, Amsterdam, The Netherlands, 2020.

Monometallic Pt catalysts used in the reduction of CO_2_ favour the formation of CO rather than CH_4_ and are attractive catalysts for the reverse water–gas shift reaction (RWGS) [121]. However, NiPt alloys can have a different performance compared to monometallic Ni and Pt. Mihet et al. [119] observed a promoting effect for CO_2_ methanation by 0.5% Pt addition on 10% Ni/Al_2_O_3_, through the enhancement of Ni dispersion and NiO reducibility. Renda et al. [33] observed a similar behaviour when 0.5% Pt was added on a 10% Ni/CeO_2_ catalyst. Interestingly, as shown in Figure 13, the increase in Pt loading above 0.5% decreased the quantity of produced CH_4_. This behaviour was attributed to the fact that Pt sites could easily dissociate CO_2_ into CO, but the further conversion of intermediate CO species took place at the nearby Ni sites. When Pt was finely dispersed, CH_4_ production was high, whereas higher Pt loadings could lead to increased CO production that blocked the active Ni methanation sites.

Kikkawa et al. [31] elucidated the promoting role of isolated Pt atoms that are finely dispersed in Ni-rich NiPt alloy nanoparticles supported on Al_2_O_3_ for the CO_2_ methanation reaction. Through Ni and Pt precursor salt impregnation and reduction, Pt atoms could dissolve into the lattice of Ni nanoparticles and were slightly negatively charged as Pt^δ-^, surrounded by Ni^δ+^ atoms. It was shown that isolated Pt^δ-^ enhanced the adsorption of intermediate CO species, while also weakening the C-O bond energy. These chemisorbed CO species on Pt^δ-^ could then be easily hydrogenated into CH_4_. As a result, Ni_95_Pt_5_ supported on Al_2_O_3_ with 5% Pt atoms in the NiPt alloy led to a higher CH_4_ formation rate and CH_4_ yield compared to the monometallic Ni catalyst and other NiPt alloys with a higher Pt content. Overall, the promoting role of isolated Pt atoms on Ni_95_Pt_5_/Al_2_O_3_ rested on them acting both as CO adsorption sites, as well as H_2_ dissociation sites.

In a later work [122], it was shown, based on in-situ FTIR observations, that, on such NiPt alloy catalysts (*iso*-Pt), the adsorbed intermediate CO species that form CH_4_ were rather bridging CO between isolated Pt atoms and nearby Ni atoms. On the monometallic Pt catalyst, strongly anchored CO species were mainly found as on-top CO (terminal mode, Pt-C≡O), which were difficult to be hydrogenated into CH_4_. These on-top CO species were then preferentially desorbed as CO rather than being converted into CH_4_ and, hence, the monometallic Pt catalyst exhibited a high CO selectivity and reverse water–gas shift (RWGS) reactivity. However, the initially formed on-top CO species over isolated Pt atoms in NiPt alloys were quickly transformed into bridging CO between these isolated Pt atoms and their neighbouring Ni atoms (μ_2_-bridging mode, Pt-(C=O)-Ni). These bridging intermediate CO species were also similar to those found in the monometallic Ni catalyst between Ni-Ni neighbours and were easily further converted into CH_4_. Therefore, it was concluded that CH_4_ (and CO) selectivity could be determined by the type of adsorbed intermediate CO species. High CH_4_ selectivity for the monometallic Ni catalyst and the NiPt alloy catalyst (*iso*-Pt) that contained isolated Pt atoms was due to the occurrence of bridging CO, while CO selectivity was drastically increased in the monometallic Pt catalyst where CO was adsorbed as on-top CO.

**Figure 14:**
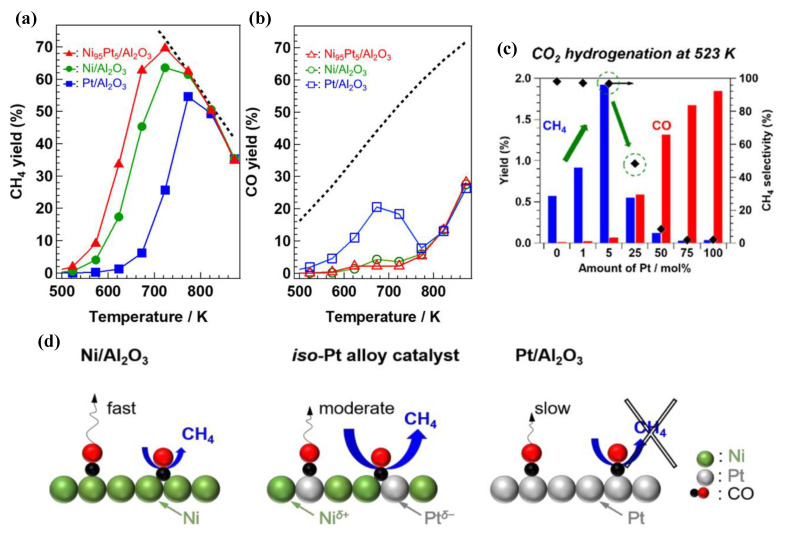
Yields of (**a**) CH_4_ and (**b**) CO following hydrogenation of CO_2_ over Ni−Pt/Al_2_O_3_, as well as (**c**) CH_4_ yield and selectivity as a function of molar Pt percentage in the NiPt alloys; m_cat_ = 100 mg, 10% CO_2_, and 40% H_2_ in He (total flow rate = 50 mL min^−1^). (**d**) Illustration of the adsorbed species and the reactions taking place over Ni/Al_2_O_3_, the *iso*-Pt catalyst, and Pt/Al_2_O_3_ during the hydrogenation of CO_2_. Reproduced with permission from [31,122]. Copyright: American Chemical Society, Washington, DC, USA, 2019, 2020.

Furthermore, the rate determining step (RDS) for CO_2_ methanation over such NiPt alloy catalysts with isolated Pt atoms was suggested to be the chemisorption of H_2_, based on kinetic studies [122]. That could be explained by the fact that although Pt sites favoured the dissociation of H_2_ at low temperatures, isolated surface Pt atoms were preferentially covered by CO rather than H_2_, due to the enhanced Pt-CO bonding. As a result, this limited availability of H_2_ dissociation sites on isolated Pt atoms became the factor that restricted the formation of CH_4_ over NiPt alloys. Therefore, the RDS for CO_2_ methanation over NiPt alloys was suggested to be H_2_ dissociation rather than H-assisted CO dissociation or hydrogenation of surface carbon species, which are commonly indicated as the RDS over monometallic Ni methanation catalysts [7,122]. The experimental results and reaction mechanism for these two works are summarized in Figure 14 [31,122].

As discussed previously with regard to Ru doping, adding Pt in Ni-based DFMs could also increase the low-temperature reducibility of NiO. The addition of 1% Pt increased the NiO reducibility by 50%, compared to the 70% increase caused by the addition of 1% Ru [104]. Employing in-situ DRIFTS, it was shown that Pt acted to further promote CO_2_ chemisorption and dissociation by forming Pt-CO species [105]. However, since Pt is not active for CH_4_ formation, these chemisorbed carbonyl intermediates were found to spill over towards Ni sites upon H_2_ inflow, where they were further hydrogenated into CH_4_. Overall, the NiRu bimetallic DFMs yielded more CH_4_ compared to the NiPt ones, since Ru sites could also participate in the CO_2_ methanation reaction [104].

In short, despite monometallic Pt catalysts being very active for the RWGS reaction, Ni-rich NiPt alloys appear to promote CH_4_ formation [31,33]. The dilution of Pt atoms over Ni metallic nanoparticles changes the CO_2_ methanation pathway, since Pt sites accommodate adsorbed CO intermediates due to their high affinity with carbonyl [31]. In general, it seems that a very low amount of Pt added in Ni catalysts, even compared to other noble metals, can lead to a significant promotion for CO_2_ methanation [33]. It should, however, be argued whether the extent of such promotion can be achieved via less expensive metals, like Ru.

### 3.4. Promotion with Pd

Pd is another noble metal with a high number of applications in heterogeneous catalysis [123,124]. Stand-alone Pd catalysts are not commonly used for CO_2_ methanation, but NiPd combinations can sometimes have a superior activity for this reaction. An example of such promotion is given by the work of Mihet et al. [119]. The addition of 0.5% Pd on 10% Ni/Al_2_O_3_ catalysts remarkably increased the low-temperature activity and the NiPd bimetallic catalyst was quite stable, outperforming other bimetallic Ni-based catalysts promoted with Pt and Rh. The addition of Pd, as well as that of other noble metals, increased the reducibility of NiO and favoured higher metal dispersion and H_2_ chemisorption. Arellano-Treviño et al. [104] also showed that Pd addition could increase NiO reducibility in DFMs, though its effectiveness for CH_4_ production is not so high compared to Ru and Pt addition.

Li et al. [32] synthesized an effective CO_2_ methanation catalyst composed of NiPd alloys supported on an SBA-15 mesoporous siliceous support. There was a strong synergetic effect between the two metals, as evidenced by H_2_-TPR, and this synergy led to the bimetallic NiPd catalysts exhibiting enhanced CO_2_ conversion compared to the monometallic Pd and Ni catalysts. The content of Ni and Pd in the alloy was also varied and it was shown that the Ni_0.75_Pd_0.25_ alloy with a Ni/Pd molar ratio of three led to the best results.

**Figure 15:**
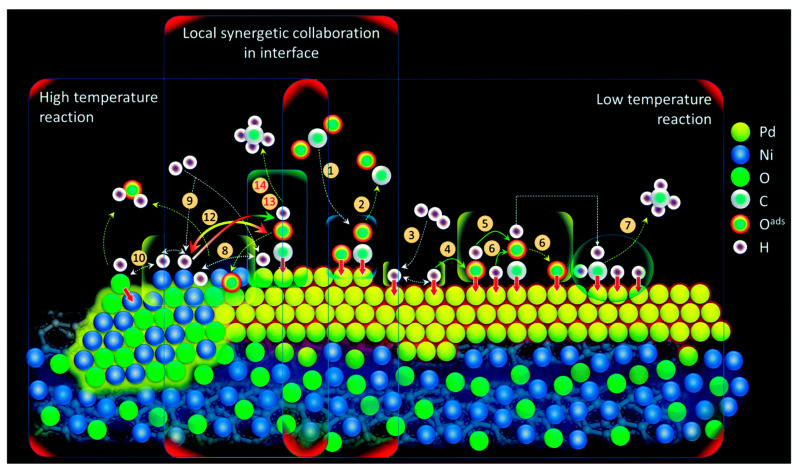
Schematic representation of the reaction coordinates in the NiO_T_Pd-T. Reproduced with permission from [125]. Copyright: Royal Society of Chemistry, London, UK, 2020.

Furthermore, Yan et al. [125] prepared a composite hierarchically structured bimetallic nanocatalyst that consisted of metallic Pd nanoclusters adjacent to NiO with local tetrahedral symmetry. The support consisted of acid-treated carbon nanotubes (CNTs) and the catalysts were further decorated with a thin tetramethyl orthosilicate (TMOS) layer. This composite NiPd bimetallic catalyst produced a maximum amount of CH_4_ and CO at 300 °C, compared to the monometallic Pd catalyst and other active catalysts with similar metal loadings that are reported in the literature. As shown in Figure 15, the higher performance of NiO_T_Pd-T catalyst was attributed to the local synergy at the interface between the Ni phase and the adjacent Pd phase, with H and CO being, respectively, chemisorbed over Ni and Pd interfacial sites. Adsorbed H atoms then helped reduce nearby NiO sites and thus increase the number of metallic Ni sites that are available for the methanation reaction.

### 3.5. Promotion with Re

Rhenium (Re) is not a very common element in heterogeneous catalysis, and it is only sometimes considered to be a noble metal. An example of its application in heterogeneous catalysis is the deoxydehydration (DODH) of biomass-derived polyols to produce adipic acid over ReO_x_ supported on ZrO_2_ [126]. There has, however, recently been a handful of reports where Re is studied as a promoter in Ni-based catalysts for methanation.

Initially, Han et al. [127] screened 63 different elements as potential promoters in Ni/Al_2_O_3_ catalysts for the methanation of CO, with the help of data mining technology. Their model predicted that promoting a Ni/Al_2_O_3_ catalyst with Re could potentially lead to the largest drop in the temperature for 50% conversion (T_50_) by 78 °C, compared to the unpromoted Ni/Al_2_O_3_. In another computational study, Yuan et al. [128] showed that the high oxygen affinity of Re can render it a favourable element to facilitate C-O bond scission during CO_2_ methanation. Their calculations indicated that isolated Re atoms on top of Ni(111) facets can accommodate O adatoms cleaved from COOH*/HCOO* and CHO*/CO* intermediates, whereas CO* and H* can be found adsorbed on the Ni(111) surface. The synergistic collaboration between the Ni surface and Re dopant atoms could promote the scission of C-O bonds, increasing CH_4_ selectivity and favouring the overall catalytic activity for CO_2_ methanation.

**Figure 16:**
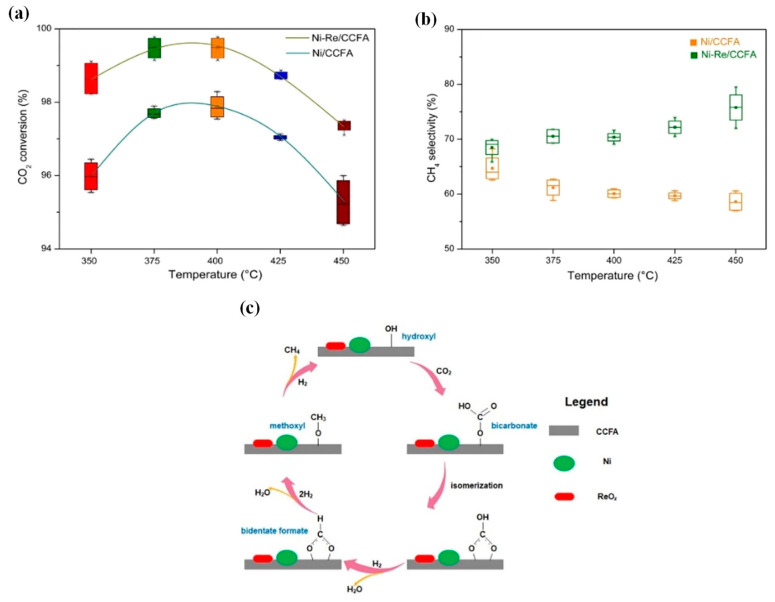
The effect of reaction temperature (**a**,**b**) on the CO_2_ conversion and CH_4_ selectivity. The conditions of reaction temperature test are H_2_:CO_2_:N_2_ = 4:1:0.5, GHSV = 2000 h^−1^ and 1 atm. (**c**) Proposed reaction mechanism of CO_2_ adsorption and methanation over reduced Ni-Re/CCFA. Reproduced with permission from [130]. Copyright: Elsevier, Amsterdam, The Netherlands, 2020.

Dong et al. [129] experimentally tested the promoting effect of Re on Ni catalysts supported on H_2_O_2_-modified manganese sand for CO methanation coupled with water–gas shift. Re was reported to decrease the activation energy for CO methanation and lead to a faster CH_4_ formation rate. Subsequently, Dong et al. [130] studied Re-promoted Ni catalysts supported on coal combustion fly ash (CCFA) derived from industrial wastes for the CO_2_ methanation reaction. The bimetallic Ni-Re/CCFA catalyst exhibited higher CO_2_ conversion and CH_4_ selectivity (99.5% and 70.3%, respectively), compared to the monometallic Ni/CCFA catalyst (Figure 16). The promoting effect of Re was attributed to the increase in Ni dispersion, as well as to the resistance of the bimetallic catalyst towards sintering and coke formation. Bicarbonate, bidentate formate and methoxyl species were detected as the reaction intermediates via in-situ DRIFTS. Table 2, below, summarises typical bimetallic Ni-based catalysts promoted with noble metals (Ru, Rh, Pt, Pd and Re) for the methanation of CO_2_.

## 4. Conclusions

The race for the development of low-cost and high-performing CO_2_ methanation catalysts stems from the need to efficiently convert excess electricity and H_2_ generated from renewables, as well as CO_2_ captured from flue gases, into a reliable energy carrier. Ni is the standard option to be used in CO_2_ methanation catalysts, due to its high activity and low cost. However, insufficient low-temperature activity and the degradation of Ni catalysts over time due to oxidation and sintering creates the need for the employment of specific metal additives to counter such drawbacks. These additives can fall in two generalized categories: other transition metals (including Fe and Co) and noble metals (including Ru, Rh, Pt, Pd and Re).

The transition metals Fe and Co offer the obvious advantage of being cheap like Ni and their similar size and electronic properties allow for their intricate interaction with the Ni primary phase and their easy dissolution into the Ni lattice, forming NiFe and NiCo alloys, respectively. The composition of the formed alloy is of great importance, since only specific bimetallic combinations can lead to an optimal CO_2_ methanation performance, especially in the case of NiFe alloys. The combined bimetallic catalysts can also offer additional advantages, such as higher stability, as well as resistance towards oxidation and sulphur poisoning.

Noble metals generally increase the reducibility and dispersion of the Ni primary phase and they can also participate in the reaction as active CO_2_ methanation phases. Stand-alone Ru catalysts are highly active for low-temperature CO_2_ methanation and the presence of Ru in bimetallic Ni catalysts as a separate monometallic phase also boosts catalytic activity. Additionally, the cost-effectiveness of Ru compared to other noble metals renders the bimetallic NiRu combinations quite popular in the field of heterogeneous catalysis. Rh and Pt can also greatly enhance the catalytic activity for CO_2_ methanation when dissolved or deposited upon Ni in small quantities. Lastly, Pd and Re have been also tested as potential promoters in Ni-based catalysts.

The assumed trade-off between cost and catalytic activity for CO_2_ methanation catalysts can be potentially overcome via the development of bimetallic Ni-containing catalysts with an optimised Ni–dopant metal synergy. Recent advances in operando spectroscopic techniques can shed light on how the reaction mechanism differs between Ni-based alloys or Ni–dopant metal interfaces and monometallic Ni, allowing for the development of catalysts with the lowest possible cost and highest possible performance.

## Figures and Tables

**Table 1:** Summary of some typical bimetallic Ni-based catalysts promoted with transition metals (Fe, Co and Cu) for the methanation of CO_2_.

Second Metal	Catalyst Composition	Preparation Method	Conditions	Performance	Comments	Ref.
Fe	10% and 30% Ni_3_Fe/Al_2_O_3_ and SiO_2_	Incipient wetness impregnation	WHSV = 32,000 mL g^−1^ h^−1^ H_2_/CO_2_ = 24/1	X_CO2_ = 35%, S_CH4_ ≈ 100% at 250 °C (30% Ni_3_Fe/Al_2_O_3_)	A 25% Fe content in the NiFe bimetallic catalysts led to the highest methanation performance. Unreduced Fe_3_O_4_ sites may have contributed by increasing CO_2_ chemisorption.	[43]
Fe	10% Ni_3_Fe/Al_2_O_3_, SiO_2_, ZrO_2_, TiO_2_ and Nb_2_O_5_	Incipient wetness impregnation	WHSV = 32,000 mL g^−1^ h^−1^ H_2_/CO_2_ = 24/1	X_CO2_ = 22.1%, at 250 °C (10% Ni_3_Fe/Al_2_O_3_)	All Ni_3_Fe bimetallic catalysts had a higher CH_4_ yield compared to the monometallic ones. The largest activity enhancement was observed for the Al_2_O_3_-supported catalyst.	[42]
Fe, Co, Cu	15% Ni_3_Fe, Ni_3_Co and Ni_3_Cu/Al_2_O_3_	Incipient wetness impregnation	WHSV = 60,000 mL g^−1^ h^−1^ H_2_/CO_2_ = 24/1	TOF_CH4_ × 10^3^ = 32.8 ± 2.3 s^−1^ at 250 °C (15% Ni_3_Fe/Al_2_O_3_)	Ni_3_Fe provided the highest TOF_CH4_ out of all bimetallic catalysts and the monometallic Ni one. Linear correlation was observed between the d-density of states at the Fermi level (N_EF_) and TOF_CH4_ based on DFT calculations.	[45]
Fe	17% Ni_3_Fe/Al_2_O_3_	Urea deposition–precipitation	WHSV = 60,000 ml_CO2_ g^−1^ h^−1^ H_2_/CO_2_ = 4/1	X_CO2_ = 78%, S_CH4_ = 99.5% at 350 °C (17% Ni_3_Fe/Al_2_O_3_)	Ni_3_Fe bimetallic catalyst with small (4 nm) and highly dispersed NiFe alloy nanoparticles provided high activity and stability. Great stability upon 45 h experiments under industrially oriented conditions.	[26]
Fe	17% Ni_3_Fe/Al_2_O_3_	Urea deposition–precipitation	H_2_/CO_2_ = 4/1 (Operando synchrotron studies)	X_CO2_ = 61%, S_CH4_ = 96% at 350 °C (17% Ni_3_Fe/Al_2_O_3_)	FeO_x_ nanoclusters were formed at the surface of NiFe alloy nanoparticles, due to oxidation of Fe^0^ in the alloy to Fe^2+^. A redox cycle between Fe^0^, Fe^2+^ and Fe^3+^ at the interface between FeO_x_ clusters and alloy nanoparticles promoted CO_2_ activation.	[49]
Fe	12% Ni and 1.2–18% Fe/MgO-Al_2_O_3_	Co-precipitation (Hydrotalcite-derived catalysts)	GHSV = 12,020 h^−1^ H_2_/CO_2_ = 4/1	Rate of CO_2_ conversion = 6.96 mmol_CO2_/mol_Fe+Ni_/s S_CH4_ = 99.3% at 335 °C (12% Ni and 1.2% Fe/MgO-Al_2_O_3_)	An Fe/(Ni + Fe) ratio of 0.1 (Ni_9_Fe_1_) provided the highest metal dispersion, the smallest size of nanoparticles and an optimum amount of surface basic sites. The corresponding catalyst had the best performance at 335 °C with the highest CH_4_ selectivity. Larger Fe loadings deactivated the catalyst for the CO_2_ methanation reaction.	[39]
Fe	20% Ni and 2–10% Fe/MgO-Al_2_O_3_	Co-precipitation (Hydrotalcite-derived catalysts)	WHSV = 43,200 ml_CO2_ g^−1^ h^−1^ H_2_/CO_2_ = 4/1	X_CO2_ = 53%, S_CH4_ ≈ 98% at 270 °C (20% Ni and 5% Fe/MgO-Al_2_O_3_)	An Fe/(Ni + Fe) ratio of 0.25 (Ni_4_Fe_1_) provided the highest CH_4_ yield at low temperatures by lowering the energy barrier for CH_4_ formation. CO_2_ hydrogenation via *HCOO (formate) intermediate was favoured over its direct dissociation to *CO	[53]
Fe	≈50% Ni and 8.5% Fe/Al_2_O_3_	Co-precipitation	WHSV = 600,000 mL g^−1^ h^−1^ H_2_/CO_2_ = 4/1	Weight time yield of CH_4_ = 30 mol h^−1^ kg_cat_^−1^ at 230 °C after 70 h aging time	Fe promoted the electronic properties of Ni through the formation of (γFe,Ni) nanoparticles. Increase in catalytic activity upon exposure to aging conditions. Fe^2+^ sites on disordered FeO_x_ were formed in-situ and promoted CO_2_ activation.	[59]
Fe, Co, Cu	30% Ni and 3% Fe, Co, Cu/ZrO_2_	Wet impregnation	GHSV = 10,000 h^−1^ H_2_/CO_2_ = 4/1 p = 0.5 MPa	X_CO2_ = 82%, S_CH4_ = 90% at 230 °C (30% Ni and 3% Fe/ZrO_2_)	Addition of 3% Fe and Co enhanced the low-temperature CO_2_ conversion. In addition, 3% Fe increased CH_4_ selectivity, but higher Fe loadings negatively influenced the methanation performance. Partly reduced Fe^2+^ sites potentially increased the dispersion of Ni and the reducibility of ZrO_2_, thus contributing to the formation of oxygen vacancies in the support.	[64]
Fe	1.51% Ni and 0.48–4.3% Fe/ZrO_2_	Incipient wetness impregnation	WHSV = 30,000 mL g^−1^ h^−1^ H_2_/CO_2_ = 2/1	X_CO2_ = 39.3%, S_CH4_ = 88.5% at 400 °C (1.51% Ni and 0.96% Fe (Ni_3_Fe_2_)/ZrO_2_)	Ni-ZrO_2_ interface was considered active for the CO_2_ methanation reaction. Fe addition up to a small amount could increase the methanation performance. Via high Fe/Ni ratios, the formed Ni-FeO_x_ interface was selective for CO, due to the weak binding of intermediate *CO species.	[65]
Fe	15% Ni and 5% Fe/surface modified activated carbon (AC)	Incipient wetness impregnation	WHSV = 60,000 mL g^−1^ h^−1^ H_2_/CO_2_ = 4/1	X_CO2_ = 77%, S_CH4_ = 98% at 400 °C (15% Ni and 3% Fe/AC-R)	Increase in activity by enhancing the surface chemistry of AC and introducing Fe. NiFe alloy formation improved the low-temperature activity, CH_4_ selectivity and stability due to the optimal CO dissociation energy.	[73]
Co	10% Ni and 3% Co/ordered mesoporous alumina (OMA)	Evaporaton-induced self-assembly (EISA)	WHSV = 10,000 mL g^−1^ h^−1^ H_2_/CO_2_ = 4/1	X_CO2_ = 78%, S_CH4_ = 99% at 400 °C (7.8% Ni and 2.2% Co/OMA)	NiCo bimetallic catalyst showed increased CO_2_ conversion and CH_4_ selectivity compared to the monometallic Ni one. Co increased H_2_ uptake. Catalysts stable at 500 °C for 60 h. NiCo alloy formation and the confinement effect of the ordered mesostructure contributed to the high stability.	[37]
Co, Fe	15% Ni and 3% Co, Fe/CeO_2_-ZrO_2_	Wet impregnation	WHSV = 12,500 mL g^−1^ h^−1^ H_2_/CO_2_ = 4/1	X_CO2_ = 83%, S_CH4_ = 94% at 300 °C (15% Ni and 3% Co/CeO_2_-ZrO_2_)	Co improved the catalytic performance for CO_2_ methanation above 250 °C. NiCo modified catalyst had great stability after many hours and could retain high activity under increased gas space velocities. Co increased Ni dispersion and could decrease coke deposition due to its redox properties.	[69]
Co, Fe, Cu	2% Co, Fe and Cu/NiO-MgO (≈35% Ni)	Sonochemical synthesis and Wet impregnation	GHSV = 47,760 h^−1^ H_2_/CO_2_ = 4/1	X_CO2_ = 90%, S_CH4_ = 99% at 400 °C (2% Co/NiO-MgO)	NiO-MgO nanocomposites prepared via the sonochemical method and promoted with 2% Co were highly active for CO_2_ methanation. The activation energy for CO_2_ methanation was lower for the Co impregnated catalysts, due to the reducing nature of Co.	[88]
Co	LaNi_1-x_Co_x_O_3_/MCF, x = 0–0.2 (≈13–16% Ni and 0–3% Co)	Citrate-assisted impregnation	WHSV = 60,000 mL g^−1^ h^−1^ H_2_/CO_2_ = 4/1	X_CO2_ = 75%, S_CH4_ = 98% at 450 °C (LaNi_0.95_Co_0.05_O_3_/MCF)	NiCo alloy nanoparticles adjacent to La_2_O_3_ and supported on mesostructured cellular foam silica (MCF) were formed after reduction. The catalyst was active and stable and 5% Co substitution in the perovskite B-site was optimal for the promotion of CO_2_ methanation.	[89]
Cu	1% Cu and 9–10% Ni/SiO_2_	Wet impregnation	WHSV = 60,000 mL g^−1^ h^−1^ H_2_/CO_2_ = 4/1	X_CO2_ = 39.5%, S_CH4_ = 44.4% at 400 °C (1% Cu and 10% Ni/SiO_2_)	Cu addition improved Ni dispersion and reducibility and NiCu alloys were formed. Cu introduction also decreased CO_2_ conversion and CH_4_ selectivity, favouring instead the production of CO via the reverse water–gas shift (RWGS) reaction.	[90]

**Table 2:** Summary of some typical bimetallic Ni-based catalysts promoted with noble metals (Ru, Rh, Pt, Pd and Re) for the methanation of CO_2_.

Second Metal	Catalyst Composition	Preparation Method	Conditions	Performance	Comments	Ref.
Ru	10% Ni and 0.5–5% Ru/Al_2_O_3_	Wet impregnation (sequential and co-impregnation)	GHSV = 9000 h^−1^ H_2_/CO_2_ = 4/1	X_CO2_ = 82.7%, S_CH4_ = 100% at 400 °C (10% Ni and 1% Ru/Al_2_O_3_)	Co-impregnation of Ni and Ru precursor salts could provide more active sites in the catalyst. The best results were achieved using 1% Ru loading. Ru and Ni were found to form separate phases and it was suggested that CO_2_ and H_2_ activation took place at Ru and Ni sites, respectively.	[30]
Ru	10% Ni and 1% Ru/2% CaO—ordered mesoporous alumina (OMA)	Evaporaton-induced self-assembly (EISA)	WHSV = 30,000 mL g^−1^ h^−1^ H_2_/CO_2_ = 4/1	X_CO2_ = 83.8%, S_CH4_ = 100% at 380 °C (10% Ni and 1% Ru/2% CaO—OMA)	A step increase in the CO_2_ conversion and CH_4_ selectivity was observed after the addition of the CaO basic promoter and the Ru second metal. Catalysts stable at 550 °C for 109 h due to the confinement effect. Ni and Ru synergy could reduce the activation energy for CO_2_ methanation.	[97]
Ru	11.1–12.7% Ni and 0.9–4.8% Ru/Al_2_O_3_-washcoated cordierite	Equilibrium adsorption (Ni) and wet impregnation (Ru)	GHSV = 104,000 h^−1^ H_2_/CO_2_ = 4/1	X_CO2_ = 55%, S_CH4_ ≈ 100% at 350 °C (12.7% Ni and 0.9% Ru/Al_2_O_3_-washcoated cordierite)	Ni was homogeneously dispersed over the structured support as small nanoparticles (2–4 nm) via equilibrium adsorption, while Ru was atomically dispersed via wet impregnation. The structured catalyst on Al_2_O_3_-washcoated monolith provided stable performance with low pressure drop under high space velocities.	[99]
Ru	30% Ni and 0–5% Ru/CeO_2_-ZrO_2_	One-pot hydrolysis of metal nitrates with (NH_4_)_2_CO_3_	GHSV = 2400 h^−1^ H_2_/CO_2_ = 4/1	X_CO2_ = 98.2%, S_CH4_ = 100% at 230 °C (30% Ni and 3% Ru/Ce_0.9_Zr_0.1_O_2_)	Ru addition on Ni/CeO_2_-ZrO_2_ could increase surface basicity and promote Ni dispersion. Thus, the formation of a NiRu bimetallic catalyst enhanced the low-temperature catalytic activity for CO_2_ methanation.	[107]
Ru	10% Ni 0.5–3% Ru/CeO_2_-ZrO_2_	Wet impregnation with different Ru precursor salts	WHSV = 60,000 mL g^−1^ h^−1^ H_2_/CO_2_ = 4/1	X_CO2_ ≈ 80%, S_CH4_ ≈ 100% at 350 °C (10% Ni and 0.5% Ru/CeO_2_-ZrO_2_)	Adding Ru in 10% Ni/CeO_2_-ZrO_2_ increased the CO_2_ methanation performance. When ruthenium acetylacetonate was used instead of ruthenium chloride as the precursor salt, the metal dispersion and catalytic activity were enhanced due to the templating effect of the precursor salt molecule.	[108]
Ru	15% Ni and 1% Ru/CeO_2_-ZrO_2_	Wet impregnation	WHSV = 24,000 mL g^−1^ h^−1^ H_2_/CO_2_ = 4/1	X_CO2_ = 53%, S_CH4_ = 93% at 350 °C (10% Ni and 0.5% Ru/CeO_2_-ZrO_2_)	The introduction of 1% Ru in 15% Ni/CeO_2_-ZrO_2_ improved the dispersion of Ni and the intrinsic activity for CO_2_ reduction. The catalyst was active for both CO_2_ methanation at 350 °C and reverse water–gas shift (RWGS) at 700 °C.	[70]
Ru	1.5% Ru/Ni	Ni deposition on Ru/SiO_2_ intermediate and then silica etching	Flow rate = 3000 mL h^−1^ H_2_/CO_2_ = 4/1	X_CO2_ ≈ 100%, S_CH4_ ≈ 100% at 200 °C (1.5% Ru/Ni)	This novel synthesis method, using an intermediate silica carrier to disperse Ru, yielded fine Ru nanoparticles supported on Ni grains. The 1.5% Ru/Ni catalyst had an oxide passivation layer and a very high low-temperature catalytic activity.	[109]
Ru	1.5% Ru/Ni nanowires (NWs)	Ni NW deposition on Ru/SiO_2_ intermediate and then silica etching	Flow rate = 3000 mL h^−1^ H_2_/CO_2_ = 4/1	X_CO2_ ≈ 100%, S_CH4_ ≈ 100% at 179 °C (1.5% Ru/Ni NWs)	When Ni nanowires were used instead of Ni powder, the higher surface area of the 1D nanostructure led to a higher CO_2_ methanation catalytic activity, with 100% CO_2_ conversion being reached at just 179 °C.	[110]
Rh, Ru	5% Ni and 0.5% Rh, Ru/CeO_2_-ZrO_2_	Pseudo sol-gel in propionic acid	GHSV = 43,000 h^−1^ H_2_/CO_2_ = 4/1	X_CO2_ = 77.8%, S_CH4_ = 99.2% at 350 °C (0.5% Rh and 5% Ni/Ce_0.72_Zr_0.28_O_2_)	Noble metal addition (Rh and Ru) increased the dispersion of Ni and the catalytic activity for CO_2_ methanation. Rh addition led to slightly better results compared to Ru.	[106]
Rh	Rh-Ni/3DOM-LaAlO_3_ (≈2% Ni and 1% Rh)	Rh wet impregnation and Ni exsolution from LaAl_0.92_Ni_0.08_O_3_	WHSV = 48,000 mL g^−1^ h^−1^ H_2_/CO_2_ = 4/1	X_CO2_ ≈ 93%, S_CH4_ high at 308 °C (Rh-Ni/3DOM-LaAlO_3_)	Rh-Ni/3DOM-LaAlO_3_ with bimetallic NiRh alloy nanoparticles was highly efficient for CO_2_ methanation. 3DOM LaAl_0.92_Ni_0.08_O_3_ perovskite was prepared via PMMA colloidal crystal templating and Rh was added via wet impregnation. Ni exsolution and NiRh alloy formation followed after reduction treatment.	[115]
Rh	1.56–1.9% Ni 0.69–1.18% Rh/Al_2_O_3_	Galvanic replacement (GR) and chemical reduction (CR)	WHSV = 48,000 mL g^−1^ h^−1^ H_2_/CO_2_ = 4/1	X_CO2_ ≈ 97%, S_CH4_ ≈ 100%, at 300 °C (1.56% Ni and 1.08% Rh/Al_2_O_3_ (GR))	RhNi/Al_2_O_3_ catalysts prepared by galvanic replacement exhibited superior CO_2_ methanation performance at low temperatures. Galvanic replacement led to Ni nanoparticles encapsulated by an atomically thin RhO_x_ shell, while chemical reduction led to a higher degree of Ni and Rh intermixing.	[117]
Pt, Ru, Rh	10% Ni and 0.5–3% Pt, Ru and Rh/CeO_2_	Wet impregnation (sequential)	WHSV = 60,000 mL g^−1^ h^−1^ H_2_/CO_2_ = 4/1	X_CO2_ = 82%, S_CH4_ ≈ 100%, at 325 °C (10% Ni and 0.5% Pt/CeO_2_)	Promotion of 10% Ni/CeO_2_ with Pt and Ru further increased the catalytic activity, while Rh led to the worst performance. The optimal Pt loading was 0.5%, while for Ru it was 1%. Pt enhanced the dissociation of CO_2_ into intermediate CO, while Ru provided additional methanation sites.	[33]
Pt	Ni_100-x_Pt_x_/Al_2_O_3_, (1 mmol Ni + Pt metal g_cat_^−1^)	Wet impregnation	WHSV = 30,000 mL g^−1^ h^−1^ H_2_/CO_2_ = 4/1	X_CO2_ = 70%, S_CH4_ = 97%, at 427 °C (Ni_95_Pt_5_/Al_2_O_3_)	Single atom alloy catalysts (SAAC) with Pt atoms dissolved into the lattice of Ni nanoparticles supported on Al_2_O_3_ were highly active for CO_2_ methanation. A Pt/(Ni + Pt) molar ratio of 5% was optimal. Isolated Pt atoms adjacent to Ni enhanced the adsorption of intermediate CO, while weakening the C-O bond energy and thus favoured the further conversion to CH_4_.	[31]
Pd, Pt and Rh	10% Ni and 0.5% Pd, Pt and Rh/Al_2_O_3_	Incipient wetness impregnation	GHSV = 5700 h^−1^ H_2_/CO_2_ = 4/1	X_CO2_ = 90.6%, S_CH4_ = 97%, at 300 °C (10% Ni and 0.5% Pd/Al_2_O_3_)	Pd and Pt addition improved the catalytic activity of 10% Ni/Al_2_O_3_, while Rh addition led to worse performance. Pd and Pt increased the dispersion and reducibility of NiO and provided active sites for H_2_ chemisorption and activation. The Pd-promoted catalyst was slightly better than the Pt-promoted one.	[119]
Pd	Ni_1-x_Pd_x_/SBA-15	NiPd nanoparticle synthesis in oil amine and impregnation on SBA-15 with hexane	WHSV = 6000 mL g^−1^ h^−1^ H_2_/CO_2_ = 4/1	X_CO2_ = 96.1%, S_CH4_ = 97.5%, at 430 °C (Ni_0.75_Pd_0.25_/SBA-15)	The synergy between Ni and Pd metals led to active NiPd alloy catalysts for CO_2_ methanation supported on mesoporous SBA-15 support. All bimetallic catalysts were better than the monometallic ones. A Ni/(Ni + Pd) ratio of 3 (Ni_0.75_Pd_0.25_) led to the best performing catalyst.	[32]
Pd	30% NiO_T_Pd-TMOS/acid-treated CNTs (Pd/Ni molar ratio = 1.5)	Ni wet impregnation on acid-treated CNTs, Pd addition with NaBH_4_ and TMOS decoration	WHSV = 100,000 mL g^−1^ h^−1^ H_2_/CO_2_ = 3/1	Methane yield: Y_CH4_ = 1905.1 μmol CH_4_ g_cat_^−1^, at 300 °C (NiO_T_Pd-TMOS/acid-treated CNTs)	The catalyst consisted of Pd nanoclusters adjacent to NiO with tetrahedral symmetry, supported on acid-treated CNTs and decorated with a layer of tetramethyl orthosilicate (TMOS). The maximum amount of CH_4_ was produced due to the Ni-Pd synergy at their interface, with H and CO being adsorbed over interfacial Ni and Pd sites, respectively.	[125]
Re	15% Ni and 1% Re/CCFA (coal combustion fly ash)	Wet impregnation (co-impregnation)	GHSV = 2000 h^−1^ H_2_/CO_2_ = 4/1	X_CO2_ = 99.6%, S_CH4_ = 70.3%, at 400 °C (NiRe/CCFA)	NiRe bimetallic catalysts were supported on coal combustion fly ash (CCFA) from industrial waste. Re addition improved Ni dispersion and the catalyst resistance towards sintering and coking, leading to better performance for CO_2_ methanation.	[130]

## Data Availability

Data sharing is not applicable to this article as no new data were created or analyzed in this study.
